# Recognition of yeast β-glucan particles triggers immunometabolic signaling required for trained immunity

**DOI:** 10.1016/j.isci.2024.109030

**Published:** 2024-01-26

**Authors:** Cian J.H. Horneck Johnston, Anna E. Ledwith, Mimmi L.E. Lundahl, Hugo Charles-Messance, Emer E. Hackett, Simon D. O’Shaughnessy, Jonah Clegg, Hannah Prendeville, John P. McGrath, Aaron M. Walsh, Sarah Case, Hollie Austen Byrne, Parth Gautam, Elaine Dempsey, Sinead C. Corr, Frederick J. Sheedy

**Affiliations:** 1 School of Biochemistry & Immunology, Trinity College, Dublin 2, Ireland; 2 School of Medicine, Trinity College, Dublin 2, Ireland; 3 School of Genetics & Microbiology, Trinity College, Dublin 2, Ireland; 4 APC Microbiome Ireland, University College Cork, Cork, Ireland

**Keywords:** Physiology, Molecular biology, Immunology

## Abstract

Fungal β-glucans are major drivers of trained immunity which increases long-term protection against secondary infections. Heterogeneity in β-glucan source, structure, and solubility alters interaction with the phagocytic receptor Dectin-1 and could impact strategies to improve trained immunity in humans. Using a panel of diverse β-glucans, we describe the ability of a specific yeast-derived whole-glucan particle (WGP) to reprogram metabolism and thereby drive trained immunity in human monocyte-derived macrophages *in vitro* and mice bone marrow *in vivo*. Presentation of pure, non-soluble, non-aggregated WGPs led to the formation of the Dectin-1 phagocytic synapse with subsequent lysosomal mTOR activation, metabolic reprogramming, and epigenetic rewiring. Intraperitoneal or oral administration of WGP drove bone marrow myelopoiesis and improved mature macrophage responses, pointing to therapeutic and food-based strategies to drive trained immunity. Thus, the investment of a cell in a trained response relies on specific recognition of β-glucans presented on intact microbial particles through stimulation of the Dectin-1 phagocytic response.

## Highlights

• *Saccharomyces cerevisiae*-derived β-glucan particles drive myeloid trained immunity
• The Dectin-1 phagocytic synapse driven by pure β-glucans particles drives training
• Phagolysosomal maturation is required for upregulation of oxidative metabolism
• *In vivo* delivery of β-glucan particles alters bone marrow myelopoiesis

## Graphical abstract

**Figure:**
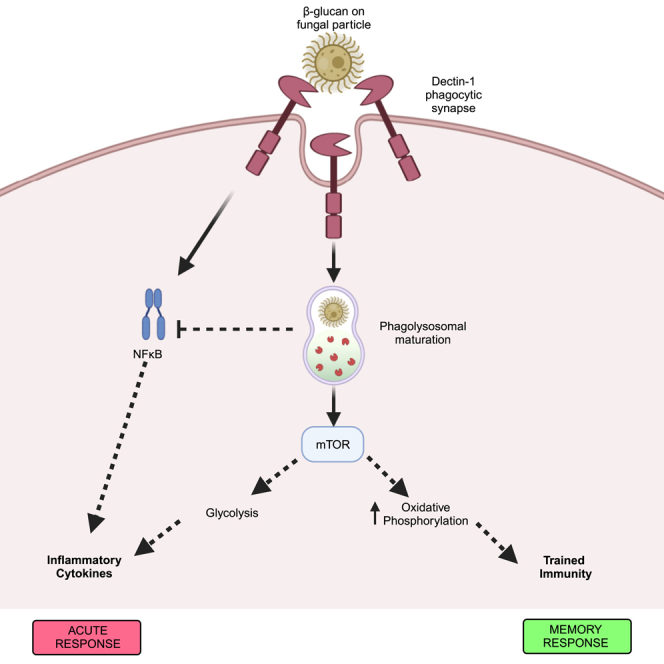

## Introduction

The importance of adaptive immune memory has been re-emphasized in light of the COVID-19 pandemic with the development of safe and effective vaccines.^1^ Recent data suggest that innate immune cells can also adapt to challenge and alter subsequent responses,^2^ an important paradigm pertaining to immune function. This has been referred to as trained immunity wherein immature cells of the myeloid lineage, for example monocytes, naive macrophages, and hematopoietic stem progenitor cells (HSPCs) have emerged as particularly amenable to this. Exposure to microbial stimuli has been shown to metabolically and epigenetically alter trained cells, such that innate immune genes are primed at the chromatin level. Thus, upon maturation and re-challenge, trained cells exhibit altered, often enhanced, responses with accelerated kinetics.^3^

β-Glucans, which have emerged as key drivers of trained immunity,^4^ comprise a heterogeneous family of structural carbohydrates with multiple biological activities.^5^ Fungal β-glucans are linked by β-1,3 and β-1,6 glycosidic bonds which make them ligands for recognition by the phagocytic C-type lectin receptor, Dectin-1 (encoded by *Clec7a*).^6^ Dectin-1 also has signaling functions and has been shown to activate nuclear factor κB (NF-κB) and pro-inflammatory gene expression through SYK/CARD9.^7^^,^^8^ However, disparate β-glucans differentially interact with Dectin-1 depending on the mode of presentation.^9^ While soluble low molecular weight (MW) β-glucans can bind Dectin-1 and drive NF-κB, recognition of larger β-glucan chains presented on intact fungal particles is required to drive surface Dectin-1 receptor localization and formation of the phagocytic synapse, linked to antimicrobial activities like reactive oxygen species.^9^ Additionally, recognition of β-glucan in some training models drives metabolic reprogramming through a PI3K/mTOR-dependent pathway, required to upregulate glycolysis.^10^ Much of this work was demonstrated using β-glucan derived from *Candida albicans*.^4^^,^^10^^,^^11^ More recent work showing *in vivo* training through bone marrow HSPC reprogramming and myelopoiesis was demonstrated following intraperitoneal (IP) injection of macrofungi (mushroom) *Trametes versicolor* or *C. albicans* β-glucan.^12^^,^^13^ Importantly, more common yeast β-glucans, particularly food-grade baking and brewer’s yeast, contain more branched, larger MW β-glucans to which various health benefits have been ascribed^14^ and their impact on trained immunity is only beginning to be considered.^15^^,^^16^^,^^17^

To determine if β-glucans from more ubiquitous fungal species like *Saccharomyces cerevisiae* modulate myeloid function through trained immunity, we employed a well-defined, intact yeast whole-glucan particle (WGP).^9^ We compared it to other β-glucans (see Table 1), in an effort to define the structural and signaling requirements for immune training in monocytes and macrophages *in vitro* and also in mice *in vivo*. Although a variety of β-glucan preparations can signal through Dectin-1 to drive pro-inflammatory responses, only relatively pure, non-soluble particulate β-glucans trigger the phagocytic synapse and internalization of the β-glucan-Dectin-1 complex, to drive metabolic reprogramming required for trained immunity. This non-soluble form of yeast WGP also enhances myelopoiesis and macrophage function when administered to mice, pointing toward therapeutic approaches to drive trained immunity in humans.

**Table 1:** β-Glucan preparations used in this study

	Fungi							Lichen	Algae	Bacterial
Source	Yeasts					Macrofungi				
	Pathogenic	Non-pathogenic				Mushroom				
Species	*C. albicans*	*S. cerevisiae*				*T. versicolor*	*S. commune*	*L. pustulata*	*L. digitata*	*A. faecalis*
Linkages	β1->3	β1->3, β1->6				β1->4, β1->3, β1->6	β1->3, β1->6	β1->6	β1->3	β1->3
Preps used in this study (Acronym)	Heat-killed *C. albicans* (HKCA)	Zymosan-A (ZYM)	depleted Zymosan-A (ZYM-d)^a^	dispersible whole-glucan particle (dWGP)	solubilized WGP (sWGP)^b^	Beta-glucan peptide (BGP)	Schizophyllan (SPG)	Pustulan (PUST)	Laminarin (LAM)	Curdlan (CURD)
Particulate	Inactivated cells	Crude ghost cell particulate	Ghost cell particulate	Ghost cell particulate	–	–	–	–	–	–
Solubility in Water	Insoluble	Insoluble	Insoluble	Insoluble	Soluble	Soluble	Insoluble	Soluble	Soluble	Insoluble
Reported MW (kDa)	∼296	–	–	∼500	Heterogeneous mix	∼100	76.8–450	∼20	3.5–7.7	53–2000
PRRs	Multiple	TLR2, TLR4, Dectin1	Dectin1	Dectin1	Dectin1(a)	Dectin1	Dectin1	Dectin1	Dectin1(a)	TLR2, Dectin1, NLRP3

a ZYM is treated with base to remove TLR2 and TLR4 ligands, yielding ZYM-d.

b dWGP is boiled in acid to break apart the whole glucan particles into soluble fragments, yielding sWGP.

## Results

### Different β-glucans lead to distinct macrophage memory responses

**Figure 1:**
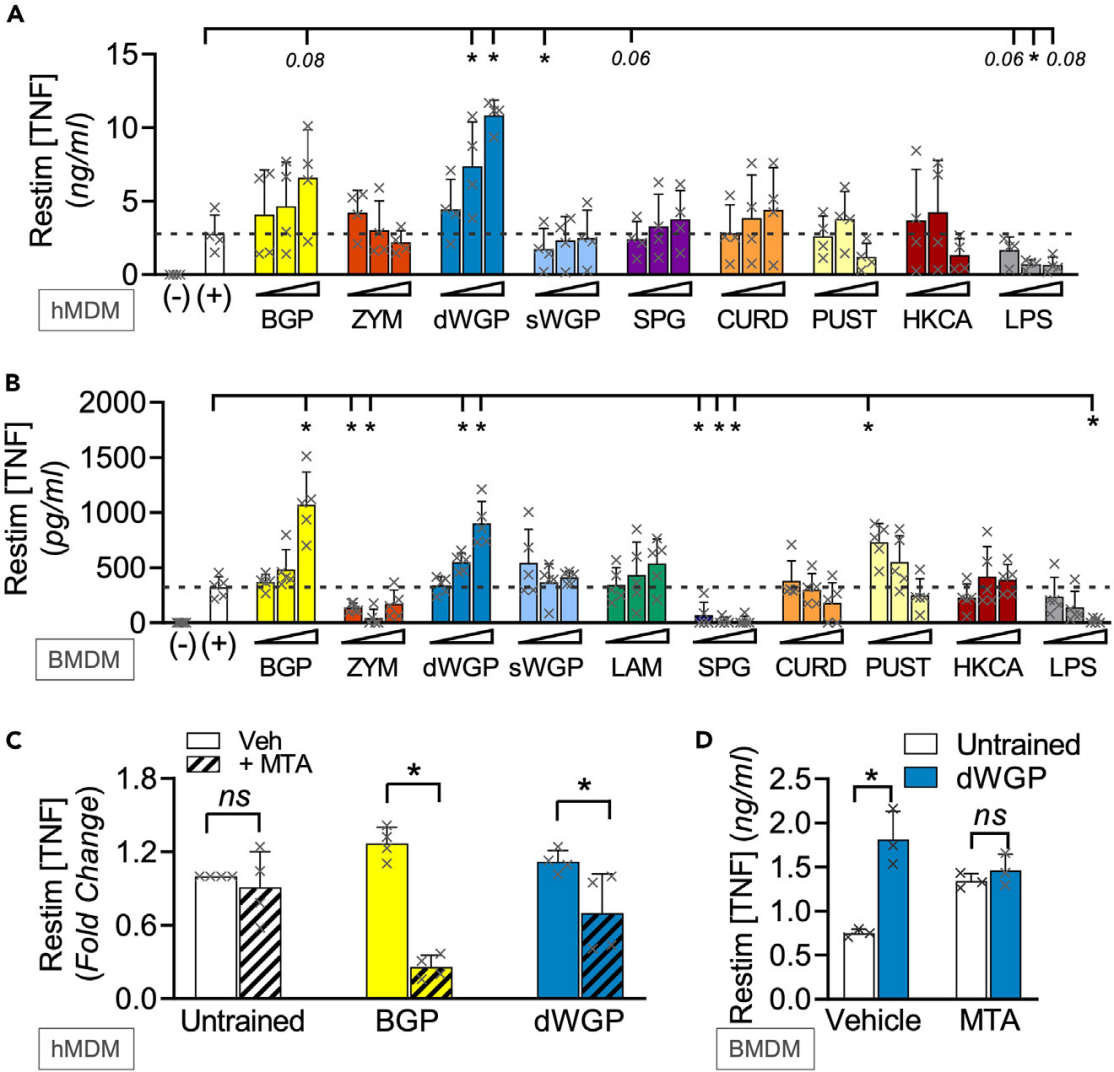
Different β-glucans lead to distinct macrophage memory responses (A–D) Training assay in hMDMs (A) or BMDMs (B) of cells trained with varying concentrations of the following β-glucans (BGP, Zymosan (ZYM), dispersible WGP (dWGP), soluble β-glucan derived from heat-treated dWGP (sWGP), Laminarin (LAM), Schizophyllan (SPG), Curdlan (CURD), and Pustulan (PUST) all at 1,10, and 100 μg/mL β-glucan, heat-killed *Candida albicans* (HKCA) at 10^4^, 10^5^, and 10^6^ cells/mL, or LPS (1, 10, and 100 ng/mL). TNF production following LPS restimulation (10 ng/mL, 6 h (A) or 3 h (B)) was measured and expressed relative to untrained (−/+) control cells. (C, D) Training assay in hMDMs (C) or BMDMs (D) exposed to 5′methylthioadenosine (MTA, 1 mM) 1 h prior to training with BGP or dWGP for 24 h, washed, matured, and restimulated with LPS (10 ng/mL, 6 h). Data are mean TNF concentration or fold-change over untrained cells ± SD for n = 4 (A, C), n = 5 (B, D) independent experiments. ^∗/#^p < 0.05, ns or indicated p ≥ 0.05 determined using multiple comparisons testing following two-way ANOVA. See also Figure S1.

In a preliminary screen, we first tested a variety of commonly available β-glucans (Table 1) for their ability to induce trained immunity in both human and mouse cells. We exposed freshly isolated human monocytes or naive mouse bone-marrow-derived macrophages (BMDMs) to similar concentrations of β-glucans for 24 h. After washing and maturing to human monocyte-derived macrophages (hMDMs) or further differentiating BMDMs, cells were restimulated with the TLR4 ligand lipopolysaccharide (LPS) and extracellular tumor necrosis factor (TNF) production measured as a readout of re-activation^11^^,^^18^^,^^19^^,^^20^^,^^21^^,^^22^ (Figures 1A and 1B). Our panel employed well-described Dectin-1 activators *T. versicolor*-derived β-glucan peptide (BGP) and *S. cerevisiae* Zymosan (ZYM; a cell wall β-glucan preparation) as positive controls,^23^ alongside similar concentrations of β-glucans derived from fungal (Schizophyllan; SPG), bacterial (Curdlan; CURD), and lichen (Pustulan; PUST) sources.^24^^,^^25^ BGP training leads to enhanced responses to restimulation in both human and mouse macrophages, consistent with earlier reports.^26^ However, unexpectedly, ZYM treatment led to impaired cytokine responses to restimulation in human and mouse cells, akin to the tolerance phenomenon^20^ when cells were treated with the negative control stimulus, LPS. A similar tolerized response was observed in SPG-treated mouse cells. The most significant and consistent training response was observed when mouse or human cells were treated with a particulate form of *S. cerevisiae* β-glucan, where the β-glucan layer of the yeast cell wall is isolated and intact forming “ghost cells,” referred to as a dispersible WGP (dWGP).^9^ A derived soluble form of the same *S. cerevisiae* β-glucan—referred to here as solubilized WGP (sWGP)—did not alter responses to restimulation. Similarly, another soluble, low MW β-glucan, laminarin,^27^ did not alter responses over untrained mouse cells. At lower concentrations, PUST drove some training in BMDMs, while CURD or heat-killed *Candida albicans* (HKCA) did not significantly alter responses in either system.

### Macrophage responses to restimulation in β-glucan-trained macrophages

Since both yeast-derived dWGP and *T. versicolor*-derived BGP were the most consistent drivers of enhanced responses across both systems, we used these to further characterize the requirements for β-glucan-induced trained immunity. The enhanced TNF production seen in both dWGP and BGP-trained hMDMs was abolished when monocytes were pre-treated with 5′methylthioadenosine (MTA) prior to β-glucan training (Figure 1C). dWGP-induced training was also not observed in BMDMs pre-treated with MTA pre-treatment (Figure 1D). This inhibition of intracellular DNA methyltransferases during the training phase confirms that these diverse β-glucans alter myeloid cells via epigenetic modification, consistent with other well-described drivers of trained immunity.^28^ To confirm that both BGP and dWGP drive innate memory responses characteristic of trained immunity seen with *C. albicans* β-glucan and other training stimuli,^21^^,^^29^^,^^30^ we profiled restimulation responses in both hMDMs (Figures S1A–S1C) and BMDMs (Figures S1D–S1F). These data show (1) enhanced and faster responses to LPS stimulation (Figures S1A and S1D), (2) that a broad range of cytokines were affected including chemokines and anti-inflammatory genes (Figures S1B and S1E), and, importantly, (3) this enhanced responsiveness was non-specific and was seen in response to a range of restimulation ligands including bacterial, viral, and fungal (Figures S1C and S1F). A broad range of cytokines (TNF, IL-1β, and IL-10) were enhanced when dWGP-trained hMDMs were stimulated with irradiated bacterial *Mycobacterium tuberculosis* H37Rv, while tolerizing with LPS dampened these responses (Figure S1G). Early containment of viable Mtb H37Ra strain was also increased in dWGP-trained macrophages (Figure S1H), although cytokine responses were not significantly increased at these times (Figure S1I). This suggests that dWGP-trained macrophages possess a superior innate anti-microbial capacity, distinct to effects on cytokine levels. Importantly, the effect of enhanced TNF production driven by TLR ligands (LPS & PAM) is also observed by intracellular cytokine staining in parallel to ELISA (Figure S1J).

### Canonical Dectin-1 signaling does not distinguish β-glucan-induced trained immunity

**Figure 2:**
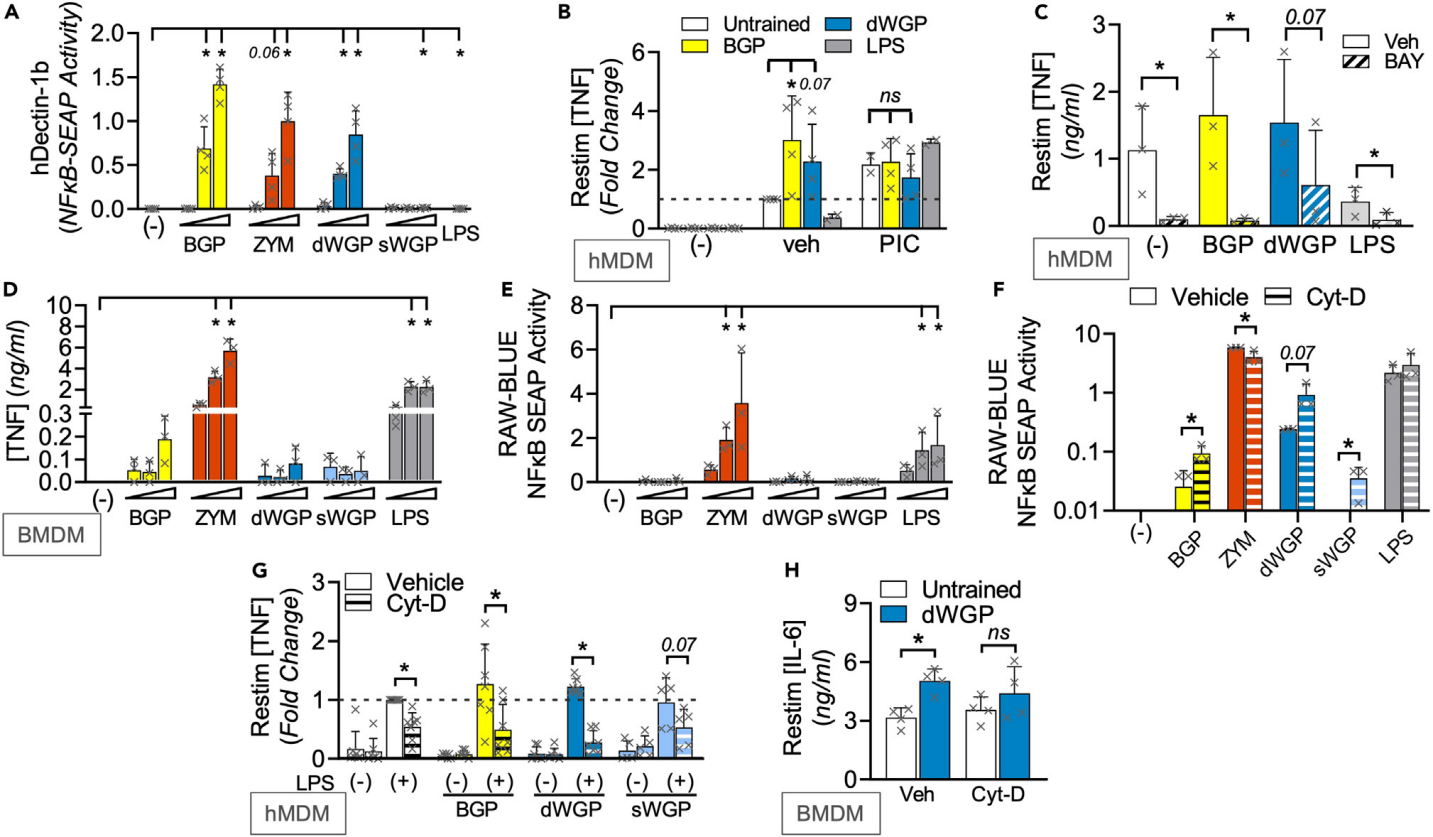
Canonical Dectin-1 signaling does not distinguish β-glucan-induced trained immunity (A) NFκB-linked SEAP activity in hDectin1b-HEK293 reporter cells incubated with the indicated β-glucans as before or LPS (10 ng/mL) or left unstimulated (−) for 6 h. (B and C) Training assay in hMDMs incubated with piceatannol (PIC; 1, 10, and 100 μM, B) or BAY-11087 (BAY, 5 μM, C) or vehicle controls (Veh) prior to training with BGP or dWGP or tolerized with LPS. Mature macrophages were restimulated with LPS (10 ng/mL, 6 h) and TNF production measured. (D) TNF production in BMDMs treated with the indicated β-glucans or LPS as before for 24 h. (E and F) NFκB-linked SEAP activity in RAW-Blue cells treated with β-glucans or left unstimulated (−) for 6 h (E) or pre-treated with cytochalasin-D (Cyt-D, 10 μM) 1 h prior to treatment with the indicated β-glucans or LPS (F). (G and H) Training assay in hMDMs (G) or BMDMs (H) pre-treated with 10 μM Cyt-D 1 h prior to β-glucans treatment. Mature macrophages were restimulated with LPS (10 ng/mL, 6 h) and TNF (G) or IL6 (H) production measured. Data are mean ± SD for n = 4 (A, B), n = 3 (C–F, H) and n = 6 (G) independent experiments. ^∗^p < 0.05, ns or indicated p ≥ 0.05 determined using multiple comparisons testing following two-way ANOVA or Student’s *t* test (F). See also Figure S2.

We next wondered what properties specific to *S. cerevisiae*-derived dWGP and *T. versicolor*-derived BGP β-glucans drove trained immunity. For simplicity, we present data for select β-glucans in main figures, with the wider β-glucan panel in Supplemental Figures. All β-glucans tested can bind Dectin-1, as revealed by HEK293-Dectin-1 overexpressing reporter cells (Figures 2A and S2A). NF-κB-linked SEAP production was driven in hDectin-1b-HEK-Blue cells to similar extents by BGP, ZYM, dWGP, and SPG, despite the heterogeneity in their long-term trained responses. HKCA, PUST, and CURD were weaker drivers of Dectin-1b, while the low MW, more soluble β-glucan preparations, sWGP, and laminarin do not activate Dectin-1b, but can trigger NF-κB activation in HEK-Blue cells overexpressing the alternate Dectin-1 isoform, Dectin-1a (Figure S2B). Thus, the ability to bind Dectin-1 does not distinguish β-glucans with the capacity to train myeloid cells (illustrated in Figure S2C). However, binding of Dectin-1 is required for training, as revealed by blocking this using pre-treatment with soluble low MW β-glucans.^9^ Pre-treatment with sWGP or laminarin blocks both dWGP and BGP-induced HEK-Dectin1b activation and monocyte training (Figures S2D–S2F). Canonical Dectin-1 signaling uses the adapter protein SYK and although SYK-independent pathways exist,^8^^,^^23^^,^^31^^,^^32^ β-glucan-driven NF-κB activation is SYK dependent.^7^^,^^8^ We confirmed this in reporter cells by blocking NF-κB activation driven by dWGP and BGP via pre-treatment with increasing concentrations of the SYK inhibitor piceatannol (PIC, Figure S2G). PIC pre-treatment blocked the enhanced trained response observed by BGP and dWGP in vehicle controls (Figure 2B). A similar result was observed when NF-κB activation downstream of dWGP was blocked, using the IKKβ inhibitor BAY 11-7082, although baseline LPS restimulation-driven TNF production was also impaired in untrained macrophages (Figure 2C), suggestive of long-term off-target effects. Despite this, the collective data suggest a model whereby canonical Dectin-1 signaling is required, but not totally sufficient to reprogram myeloid responses to restimulation.

Therefore, we examined NF-κB-dependent processes in myeloid cells after β-glucan exposure, namely examining inflammatory cytokine production. 24 h treatment of BMDMs with most β-glucans drives minor TNF production relative to the TLR4 agonist, LPS (Figures 2D and S2H). However, both ZYM and SPG drive similar TNF levels as LPS. Similar results were seen when other inflammatory cytokines were measured including IL6 and IL10, with no detectable IL-1β or the NFAT-dependent IL2 (Figure S2I). Additionally, transfer of supernatants from dWGP-treated monocytes to naive cells does not confer enhanced responsiveness to re-activation (Figure S2J), ruling out a role for a secreted NF-κB-dependent factor actively driving training. On the other hand, the ability to drive potent cytokine responses seemed linked to the development of tolerance-like phenotypes in long-term training assays (LPS, ZYM, and SPG).

We utilized the phagocytic NF-κB reporter cell line, RAW-Blues to examine this in more detail. As before, ZYM and SPG drove significant NF-κB activity in RAW-Blues, similar to LPS (Figures 2E and S2K). While other β-glucans, including dWGP and BGP, drove minimal NF-κB activation. Camilli et al.^33^ reported similar observations examining inflammatory cytokine production in DCs, where β-glucans presented on particles preferentially trigger internalization via phagocytosis and thereby limit canonical Dectin-1 signaling. When we used cytochalasin-D (Cyt-D) pre-treatment to block phagocytosis in RAW-Blue cells, we observed increased NF-κB activation by both dWGP and BGP (Figure 2F). We also observed enhanced TNF production in dWGP-treated monocytes after Cyt-D pre-treatment (Figure S2L), supporting the notion that these β-glucans are rapidly phagocytosed which limits inflammatory activation. This also impacts their ability to train, since Cyt-D pre-treatment in hMDMs and BMDMs abolishes the enhanced response over untrained cells seen with dWGP and BGP (Figures 2G, 2H, and S2M). Thus, phagocytosis and internalization of β-glucan/Dectin-1 complexes limits inflammatory activation and favors long-term training. We therefore sought to identify additional intracellular pathways and processes impacted by β-glucan drivers of training.

### β-Glucans drive intracellular metabolic reprogramming for training

**Figure 3:**
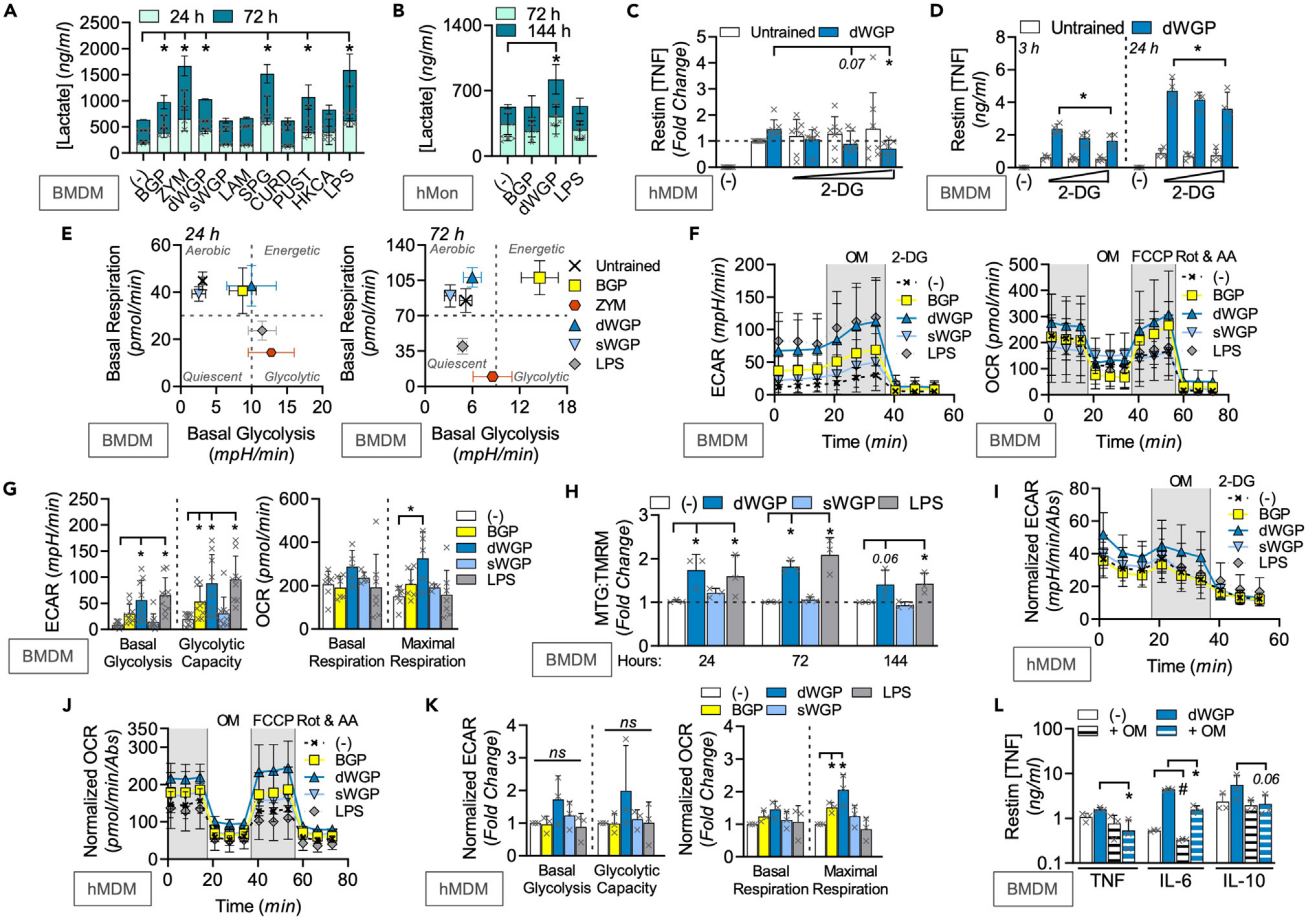
β-Glucans drive long-term metabolic reprogramming for training (A and B) Extracellular lactate in BMDMs treated with indicated β-glucans (100 μg/mL) or LPS (10 ng/mL) for 24 h and measured between 24 and 72 h (A) or human monocytes (hMon) trained with dWGP or BGP (10 μg/mL) or LPS (100 ng/mL) for 24 h and measured between 72 and 144 h (B). (C and D) dWGP training assay in hMDMs pre-treated with 2 deoxyglucose (2DG, 1, 5, and 10 μM, 1 h before dWGP, C) or BMDMs pre-treated with 25 μM 2DG (D). TNF production was measured 6–24 h after restimulation with LPS (10 ng/mL). (E) Energy plots for BMDMs treated with indicated β-glucans (100 μg/mL) or LPS (10 ng/mL) between 24 and 72 h based on measurements of basal glycolysis or respiration rates. (F and G) Extracellular flux analysis in BMDMs treated with BGP, dWGP or sWGP (100 μg/mL) or LPS (10 ng/mL) for 72 h. Glycolytic traces based on extracellular acidification rates (ECAR) after inhibitor addition (OM; oligomycin and 2DG) or respiration traces based on oxygen consumption rates (OCR) after inhibitor addition (OM, FCCP, and Rot & AA; rotenone + antimycin A) shown in F. Basal and maximal rates shown in G. (H) BMDMs were treated with dWGP or sWGP (100 μg/mL) or LPS (10 ng/mL) between 24 and 144 h and mitochondrial function measured by flow cytometry using the ratio of mitotracker green (MTG, mitochondrial mass) to tetramethylrhodamine methyl ester (TMRM, mitochondrial activity). (I–K) Extracellular flux analysis in hMDMs after training with BGP, dWGP or sWGP (10 μg/mL) or LPS (10 ng/mL) for 120 h. Normalized glycolytic traces (I) and respiration traces (J) are shown after inhibitor addition. Basal and maximal rates shown in K. (L) dWGP training assay in BMDMs pre-treated with oligomycin (+OM, 20 μM). The indicated cytokines were measured after LPS restimulation (10 ng/mL, 6–24 h). All data are mean ± SD for n = 3 (A, H–L), n = 7 (C), n = 4 (D), n = 6 (E) or n = 9 (F, G) independent experiments. ^∗/#^p < 0.05, ns or indicated p ≥ 0.05 determined using multiple comparisons testing following one or two-way ANOVA. See also Figure S3.

*C. albicans*-derived β-glucan has been shown to drive significant metabolic reprogramming in trained monocytes which is linked to the epigenetic modifications required for enhanced macrophage responsiveness.^10^^,^^34^ In particular, an upregulation of cytosolic glycolysis driven by HIF1α emerged as a key signal activated through a Dectin-1/PI3K/mTOR pathway.^10^^,^^22^ We measured the ability of a range of β-glucans to drive glycolysis in trained mouse BMDMs and human monocytes by measuring extracellular lactate production over time (Figures 3A, 3B, and S3A). We found that many of our β-glucan preparations drove significant lactate production including BGP, dWGP, ZYM, and SPG in BMDMs (Figure 3A), as did dWGP and ZYM in human monocytes (Figures 3B and S3A). Blocking the switch to glycolysis by targeting hexokinase with 2-deoxyglucose abolished the ability of dWGP to train hMDMs (Figure 3C), yet only slowed the kinetics of the enhanced TNF response in BMDMs (Figure 3D) and had no effect on enhanced IL6 or IL10 production (Figure S3B). These data suggest that like NF-κB, induction of glycolysis is required, yet not sufficient for training. We therefore extended our metabolic profiling in β-glucan-treated cells and used extracellular flux analysis of naive BMDMs to allow this (Figures S3C–S3E).

Similar to lactate production, basal extracellular acidification rates (ECARs) are rapidly increased (24 h) in BMDMs treated with dWGP, BGP, ZYM, and SPG, although they drop at later times (72 h) for ZYM and SPG, as revealed when glycolytic capacity is measured (Figure S3C). At the same time, ZYM and SPG downregulate oxygen consumption rates (OCRs) similar to LPS treatment, with significant impairments in maximal respiration rates (Figure S3C). Despite this, BGP and dWGP-treated cells increase OCR and maximal respiration capacity after 72 h and when plotted on an energy plot based on basal metabolic rates, become more aerobic and energetic, while ZYM-treated cells become more quiescent over time (Figure 3E). Thus, β-glucans associated with training maintain and upregulate levels of oxidative phosphorylation (ox-phos), while tolerance-associated stimuli downregulate ox-phos.

Examining the metabolic alterations associated with training in more detail (Figures 3F and 3G), dWGP emerges as a strong driver of overall metabolic reprogramming with significantly increased basal glycolysis, glycolytic capacity, and maximal respiration rates, and a trend toward increased basal OCR (Figure 3G). Importantly, the characteristic downregulation of ox-phos observed in tolerance (LPS) does not occur. To confirm these findings, we employed analysis of mitochondrial function by flow cytometry of β-glucan-treated BMDMs (gating shown in Figure S3F). Unlike LPS, which drives both increased mitochondrial membrane potential and mass (measured by tetramethylrhodamine and mitotracker green, respectively),^35^ dWGP treatment specifically upregulates mitochondrial activity but not mass (Figures 3H and S3G). We confirmed similar effects in human monocytes using extracellular flux analysis, which revealed a stronger increase in baseline OCR and maximal respiration rates in hMDMs trained with dWGP and BGP, than changes observed in glycolytic rates (Figures 3I–3K). Thus, while β-glucan recognition may drive glycolytic reprogramming characteristic of activation of most PRRs, β-glucans associated with training have the capacity to maintain oxidative metabolism. Importantly, this property distinguishes their training potential. To confirm the importance of this for trained immunity, we targeted the electron transport chain with oligomycin prior to dWGP treatment. This temporary inhibition of oxidative phosphorylation significantly impaired the trained response when TNF and IL6 production was measured alongside blunted IL10 production (Figure 3L).

### mTOR-independent remodeling of TCA

**Figure 4:**
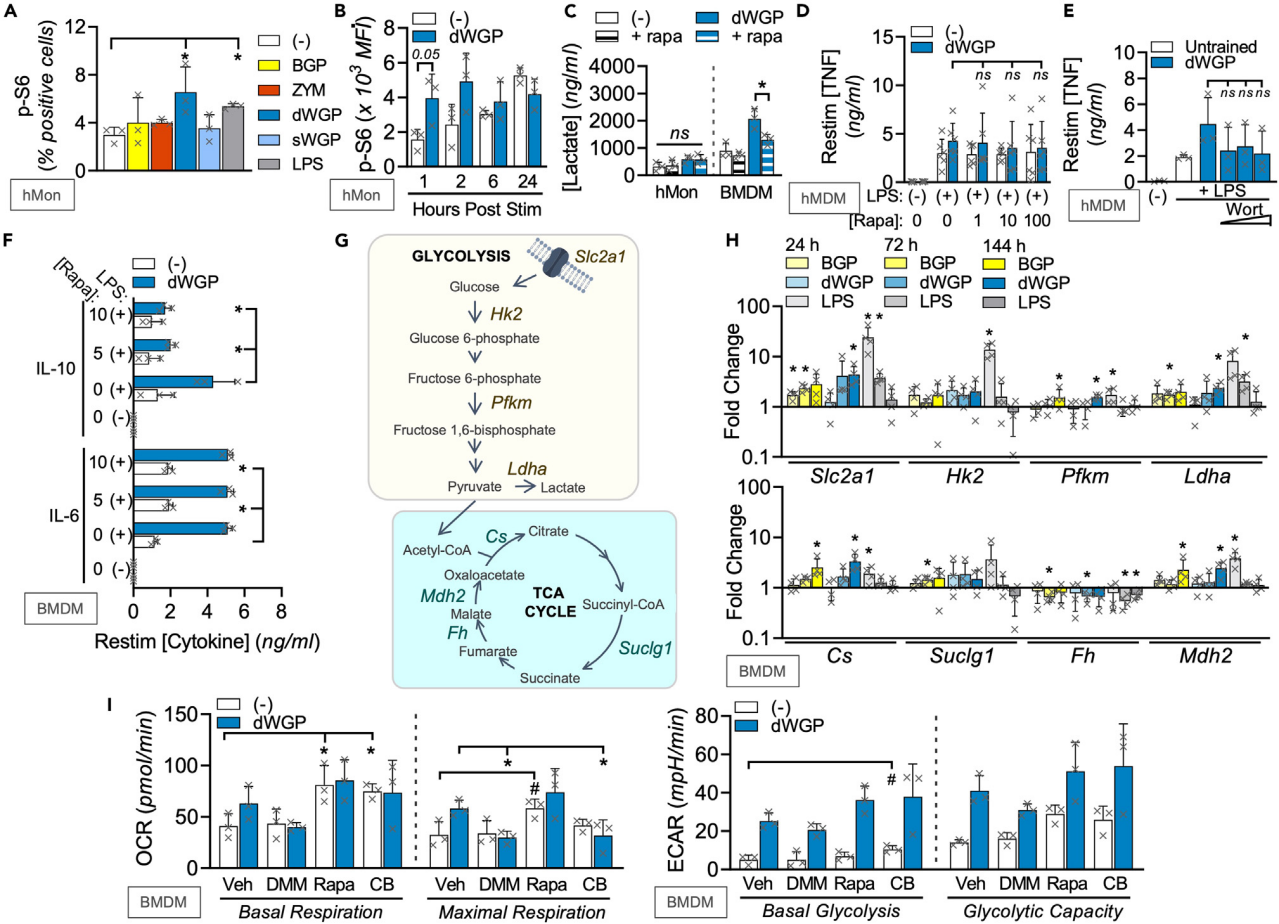
mTOR-independent remodeling of TCA during dWGP training (A) Phospho-S6 (p-S6) activation was measured by flow cytometry of human monocytes (hMon) after stimulation with the indicated β-glucans (10 μg/mL) or LPS (10 ng/mL) or left unstimulated (−) for 1 h. (B) p-S6 activity in hMons treated with dWGP (10 μg/mL) or left untreated (−) between 1 and 24 h. (C) Lactate production in hMons or BMDMs pre-treated with rapamycin (Rapa, 10 nM) after training with dWGP (72 h). (D and E) dWGP training assays in hMDMs pre-treated with Rapa (10 nM, D) or Wortmannin (Wort between 0.1, 1, and 10 μM, E) and TNF production measured after LPS restimulation (10 ng/mL, 6 h). (F) dWGP training assay in BMDMs pre-treated with Rapa (10 nM). IL10 and IL6 production were measured after LPS restimulation (10 ng/mL, 24 h). (G and H) mRNA expression of the indicated genes (shown in G), from BMDMs post-stimulation with BGP, dWGP or sWGP (100 μg/mL) or LPS (10 ng/mL) for 24, 72, and 144 h. Genes measured by qPCR are expressed relative to unstimulated controls. (I) Extracellular flux analysis in BMDMs pre-treated with Rapa (10 nM, 1 h), CB-839 (CB; 1 μM, 6 h), and di-methyl-malonate (DMM, 10 mM, 3 h) or vehicle controls and subsequently treated with dWGP (100 μg/mL) for 24 h. Basal and maximal glycolytic and respiration rates were calculated as before. All data are mean ± SD for n = 3 (A–C, E, F, I), n = 4 (H) and n = 6 (D) independent experiments. ^∗/#^p < 0.05, ns or indicated p ≥ 0.05 determined using multiple comparisons testing following one or two-way ANOVA. See also Figure S4.

We set out to uncover regulators of the metabolic reprogramming driven by β-glucan associated with training responses. *C. albicans* β-glucan drives glycolytic reprogramming via a PI3K/mTOR axis.^22^ We thus measured phosphorylation of the mTOR substrate S6 in human monocytes (Figure S4A) and found that dWGP is a strong driver of this response, alongside PUST (Figures 4A and S4B). Examining dWGP-mediated S6-phosphorylation in more detail, we found this occurs in a rapid and rapamycin-dependent manner indicative of mTOR activation, similar to LPS treatment and is specific to dWGP particles, since sWGP cannot do this (Figures S4C–S4E). Although this activity occurs rapidly post-treatment (1–2 h), it is maintained up to 24 h post-treatment when untreated monocytes also upregulate mTOR as part of the differentiation process (Figure 4B). This early mTOR activity promotes β-glucan-induced glycolysis, since pre-treatment of dWGP-trained BMDMs with rapamycin limits extracellular lactate accumulation (Figure 4C). Despite this, rapamycin pre-treatment has a minimal effect on β-glucan-induced trained responses, with no significant changes seen in TNF production after LPS restimulation in hMDMs (Figure 4D) or with wortmannin treatment to block PI3K (Figure 4E). Similarly, the enhanced TNF or IL6 response seen in dWGP-trained BMDMs was not altered by rapamycin treatment (Figures 4F and S4F), although enhanced IL-10 production appeared sensitive to mTOR inhibition (Figure 4F). Thus, although dWGP can drive rapid mTOR activation and glycolytic reprogramming, this does not link to long-term metabolic reprogramming required for trained immunity.

We thus examined other metabolic processes in β-glucan-trained cells and examined a range of metabolic genes (Figure 4G). Similar to its effect on macrophage bioenergetics, LPS treatment drove an early upregulation of a variety of rate-limiting glycolytic genes like *Slc2a*1, *Hk2*, *Pfkm*, and *LdhA* by 24 h,^36^ but which decreased at later times post treatment (between 72 and 144 h post-treatment, Figure 4H). Intriguingly, β-glucan treatment leads to a slower but significant upregulation of the same glycolytic genes, but also an upregulation in many of the tricarboxylic acid (TCA)-associated genes which are downregulated by LPS including *Cs*, *Suclg1*, and *Mdh2*, with the exception of *Fh* mRNA (Figure 4H). These data support the notion that β-glucans associated with training enhance the overall metabolic and energetic status of the macrophage through *bona fide* metabolic reprogramming. It also highlights a critical role for TCA-cycling in β-glucan training. This has recently been linked to the epigenetic requirements for β-glucan training.^37^ We thus set out to more specifically target ox-phos-associated TCA using di-methyl-malonate (DMM) which blocks succinate dehydrogenase/complex 2 on the electron transport chain.^35^ Strikingly, the upregulation ox-phos characteristic of dWGP-trained cells was abolished by 3 h DMM pre-treatment with significant impairment of maximal respiratory rates (Figure 4I). Importantly, rapamycin pre-treatment did not achieve this, nor did any of these inhibitors affect glycolysis. However, inhibition of glutaminase using the small molecule CB-839 led to similar effects on maximal respiration as DMM pre-treatment (Figure 4I). These data suggest that in the face of significant aerobic glycolysis, lactate accumulation and reduced pyruvate availability in β-glucan-stimulated macrophages, an ability to modulate TCA through anaplerotic pathways, including glutaminolysis, supports intracellular processes required for training.

### Phagocytosis of β-glucans leads to intracellular metabolic reprogramming

**Figure 5:**
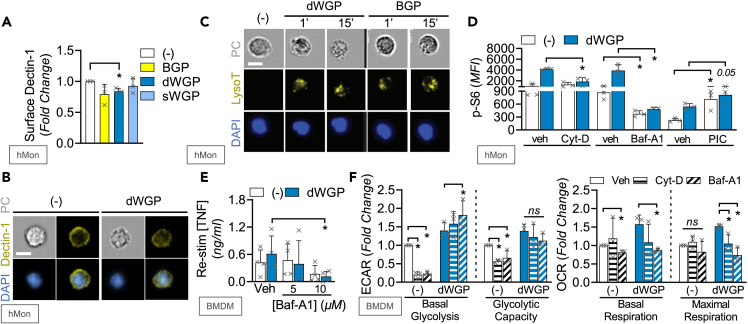
Phagocytosis of β-glucans leads to intracellular metabolic reprogramming (A and B) Surface Dectin-1 staining in human monocytes (hMon) treated with the indicated β-glucan (10 μg/mL) for 15 min measured by flow cytometry (A) or combined image-stream cytometry (B). (C) Lysotracker (LysoT) staining of permeabilized hMons after treatment with dWGP or BGP (10 μg/mL, 1–15 min) measured by combined image-stream cytometry. (D) p-S6 activity in hMons pre-treated with cytochalasin D (CytD, 100 μM), bafilomycin A1 (bafA1, 10 μM), piceatannol (PIC, 30 μM), or vehicle controls (veh) for 15 min prior to stimulation with dWGP (10 μg/mL) for 2 h. (E) dWGP Training Assay in BMDMs pre-treated with 10 μM Baf-A1 for 15 min. TNF production was measured 6 h after restimulation with LPS (10 ng/mL). (F) Extracellular flux analysis in BMDMs pre-treated with Baf-A1 (10 μM) or Cytochalasin-D (Cyt-D, 10 μM) and treated with dWGP (100 μg/mL) for 24 h. Basal and maximal glycolytic and respiration rates were calculated as before. All data are mean ± SD for n = 3 (A, D, F) or n = 4 (E) independent experiments or representative images. ^∗^p < 0.05, ns or indicated p ≥ 0.05 determined using multiple comparisons testing following one or two-way ANOVA. Scale bar: 7 μm. See also Figure S5.

Earlier data suggest that internalization of BGP and dWGP limits inflammatory activation and thus promotes training (Figure 2). However, it was not established if this process promotes metabolic changes or what characteristics of these β-glucans permitted this. Measuring cell surface Dectin-1 expression by flow cytometry, we found that dWGP led to loss of Dectin-1 surface expression shortly after treatment (15 min, Figures S5A and 5A), which is dependent on phagocytosis since pre-treatment with Cyt-D blunts this effect (Figure S5B). Decreased surface Dectin-1 in dWGP-treated monocytes is also observed using cell stream imaging of single cells (representative images shown in Figure 5B). The loss of surface Dectin-1 is not driven by sWGP and occurs less efficiently and with slower kinetics in BGP-treated cells (Figures 5A, S5B, and S5C). Consistent with increased phagocytosis,^31^ rapid lysosomal acidification is observed in dWGP-treated monocytes using Lysotracker staining in cell stream imaging (Figure 5C), which again occurs with slower kinetics in BGP-treated monocytes. Taken alongside the effect of Cyt-D on trained responses (Figure 2H), these data suggest that dWGP drives rapid internalization of Dectin-1/β-glucan complexes to promote intracellular processes required for training.

Since dWGP preferentially drove rapid S6 phosphorylation, we examined the impact of phagocytosis-linked processes on this. Blocking phagocytosis with Cyt-D pre-treatment blocked the activation of pS6 seen in dWGP-treated cells, as did blocking lysosomal acidification using bafilomycin-A1 (Baf-A1, Figure 5D). Baf-A1 pre-treatment also blocked the enhanced response seen in dWGP-trained BMDMs (Figure 5E) and hMDMs (Figure S5D). Interestingly, blocking canonical Dectin-1 signaling by targeting SYK activation with PIC did not affect mTOR activation (Figure 5D), despite blocking canonical TNF production (Figure S5E). These data suggest a bifurcation in Dectin-1 signaling from membrane-driven canonical NF-κB signaling and lysosomal-driven metabolic reprogramming required for trained immunity. We thus examined the effect of phagolysosomal maturation on WGP-induced metabolic reprogramming. Although both Cyt-D and Baf-A1 had major impacts on baseline ECAR, they did not block the upregulation in glycolytic rates seen with dWGP (Figure 5F). These treatments alone did not alter baseline OCR, yet significantly attenuated the ability of dWGP to upregulate oxidative metabolism in BMDMs, with impaired basal respiration and attenuated maximal respiratory rates (Figure 5F).

### Soluble yeast β-glucan molecules cannot trigger trained immunity

**Figure 6:**
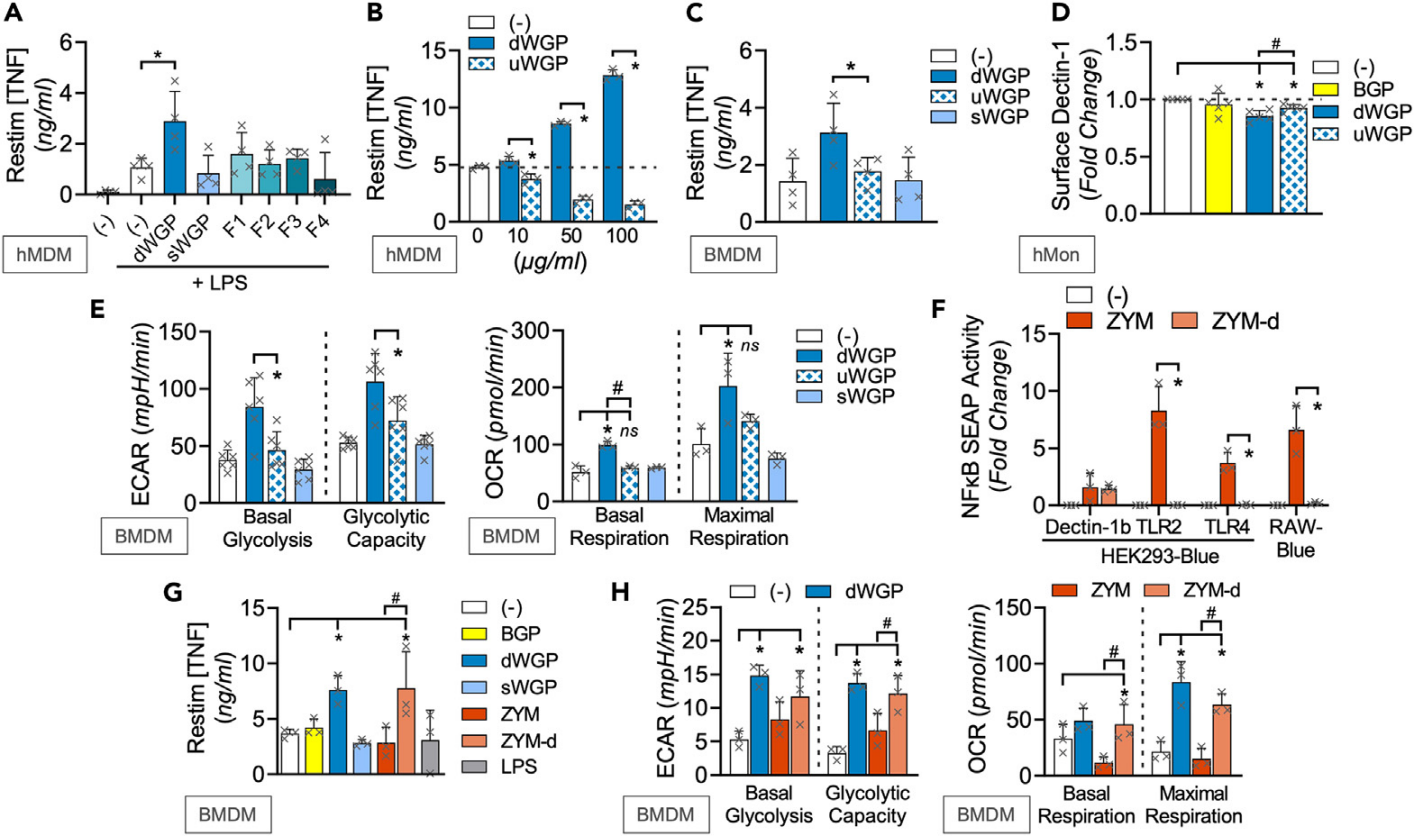
Phagocytosis of intact and pure yeast β-glucan particles drives trained immunity (A) Training assay in hMDMs trained with dWGP, sWGP, or the indicated soluble fractions F1-F4 (10 μg/mL) or left untrained (−). TNF production was measured 24 h after restimulation with LPS (10 ng/mL). (B and C) Training assay in hMDMs (B) or BMDMs (C) trained with dWGP or unsonicated WGP (uWGP) between 10 and 100 μg/mL. TNF production was measured 6 h after restimulation with LPS (10 ng/mL). (D) Surface Dectin-1 staining in human monocytes (hMon) incubated with the indicated β-glucan (10 μg/mL) for 15 min and measured by flow cytometry. (E) Extracellular flux analysis in BMDMs treated with the indicated WGPs (100 μg/mL) for 72 h. Basal and maximal glycolytic and respiration rates were calculated as before. (F) NFκB-linked SEAP activity in the indicated reporter cells after treatment with crude Zymosan (ZYM) or depleted Zymosan (ZYM-d) at 100 μg/mL for 6 h. (G) Training assay in BMDMs trained with the indicated β-glucans (100 μg/mL). TNF production was measured 6 h after restimulation with LPS (10 ng/mL). (H) Extracellular flux analysis in BMDMs treated with the indicated β-glucans (100 μg/mL) for 72 h. All data are mean ± SD for n = 4 (A, C), n = 3 (B, D–H) independent experiments. ^∗/#^p < 0.05, ns p ≥ 0.05 determined using multiple comparisons testing following one-way ANOVA or Students t tests (B). See also Figure S6.

The *S. cerevisiae* yeast-derived β-glucan particle dWGP thus emerged from our studies as a rapidly phagocytosed β-glucan capable of reprogramming cellular metabolism to favor trained immunity. However, the solubilized equivalent of the same β-glucan—which is prepared by degradation of dWGP, resulting in a heterogeneous mixture of β-glucan strands of various MWs that lack the intact single-cell structure of dWGP—does not trigger internalization, metabolic reprogramming, or training (Figures 2, 3, and 4). To determine if this was a property of MW of the β-glucan chains in these preparations, various fractions of bulk sWGP were prepared and tested alongside dWGP and sWGP (Figure S6A). Intriguingly, lower MW fractions (F1; >100 kDa & F2; 100–400 kDa) of sWGP had the strongest capacity to bind the Dectin-1b isoform in HEK293-reporter assays and as fractions increase in MW (F3->F4), this property decreases (Figure S6B). The sWGP preparation itself does not activate Dectin-1b but can bind Dectin-1a (Figure S2B). Despite this capacity to bind Dectin-1b, neither of these MW fractions displayed training in hMDM assays (Figure 6A). Thus, MW and β-glucan chain size does not explain the capacity of dWGP to train. We began to consider the particulate nature of dWGP, which previous work has demonstrated is crucial for Dectin-1 clustering and phagocytosis.^9^ Similarly sized 3 μm aminated polystyrene particles (PS) cannot drive lactate production in human monocytes similar to dWGP (Figures S6C–S6E). However, conjugating sWGP to these particles via 1,1′ carbonyldiimidazole conferred the capacity to upregulate glycolysis, significantly more than sWGP or PS particles alone (Figure S6E). This suggests that recognition of β-glucan presented on microbial-sized particles is key for both internalization and metabolic reprogramming associated with training.

### Phagocytosis of intact and pure yeast β-glucan particles drives trained immunity

The phagocytic synapse model suggests that while the presence of β-glucan can bind Dectin-1 to initiate signaling, particles which mimic fungal infection are required to cluster Dectin-1 receptors on the cell surface and trigger internalization and phagocytosis.^9^ When preparing dWGP to generate “dispersible” particles representing intact ghost yeast cells, the WGP preparation is sonicated to declump aggregates (Figure S6F). Interestingly, we noted that unsonicated WGP (uWGP), while it can bind and trigger Dectin-1b in HEK292-reporter cells similar to other β-glucan particles (dWGP, ZYM, and HKCA, Figure S6G), does not train hMDM in the same way observed for dWGP (Figure 6B). Similar effects were observed in BMDMs (Figure 6C). Thus, the presentation of β-glucan on intact single dWGPs is key for recognition and accordingly, uWGP does not trigger similar loss of cell surface Dectin-1 expression in monocytes (Figure 6D) indicative of its inability to trigger phagocytosis. As a consequence, the metabolic reprogramming observed for dWGP does not occur, with baseline extracellular lactate production seen after uWGP treatment of human monocytes (Fig S6H). In mouse cells, no significant enhancement of metabolic parameters (basal ECAR, basal OCR, glycolytic capacity, and maximal respiration) is observed with uWGP, unlike dWGP (Figure 6E). Thus, recognition and phagocytosis of intact yeast β-glucan particles is required for the metabolic reprogramming of cells.

Our data up to now, however, illustrate that another well-characterized β-glucan particle, also derived from *S. cerevisiae*, ZYM, does not drive trained immunity. In fact, it tolerized cells and drove metabolic reprogramming toward a quiescent state similar to LPS (Figures 1B and 3E). We thus examined the purity of our ZYM preparation and although it drives similar Dectin-1b activation in HEK293 reporter assays (Figures S2A and S2B), it also triggered both TLR4 and TLR2 activity (Figure S6I), a property not observed with dWGP or many other β-glucans. SPG, however, which also drove tolerance responses and downregulates oxidative metabolism (Figures 1, 2, and 3), also triggered TLR2 and TLR4 (Figure S6I). Crude ZYM is known to contain mannans and other lipoproteins which are putative TLR ligands.^23^^,^^38^ We thus obtained a more pure form of ZYM, depleted of these ligands; ZYM-depleted (ZYM-d).^39^^,^^40^ This preparation bound Dectin-1b similar to ZYM, but did not trigger TLR2, TLR4, or NF-κB activation in RAW-Blues (Figure 6F), suggesting it is rapidly phagocytosed. In mouse BMDM training assays, ZYM-d also drove a significantly higher TNF response after restimulation with LPS than the tolerized response seen with ZYM alone (Figure 6G). Although ZYM-d drove similar levels of glycolysis as crude ZYM when ECAR is measured via metabolic flux analysis, there was a strong upregulation of oxidative metabolism with increased basal OCR (Figure 6H). This is consistent with its ability to drive enhanced responses to restimulation over untrained cells seen in hMDM and BMDM training assays (Figures S6J and S6K). Thus, recognition of pure, non-contaminating β-glucan on intact particles is key for phagocytosis and intracellular metabolic changes which underly trained immunity.

### dWGP drives myeloid bone marrow reprogramming

HSPCs^41^ have emerged as sensitive to systemic delivery of training stimuli through various methods, including intravenous delivery of BCG,^42^ IP injection of *C. albicans* β-glucan,^13^ or hypercholesterolemia-induced NLRP3 inflammation driven by western diet feeding.^43^ In particular, the resulting inflammation leads to an increase in total bone marrow c-Lin^-^, ckit^+^, Sca-1^+^ cells (LKS+) HSPC numbers with an increasing skewing in the ratio of multipotent progenitors (MPPs) toward myeloid-committed MPP3 and away from the more dominant lymphoid MPP4 cells. We thus undertook to determine if similar long-term immune training can be observed *in vivo* in response to yeast β-glucan, by examining HSPC progenitor cells in mouse bone marrow.

**Figure 7:**
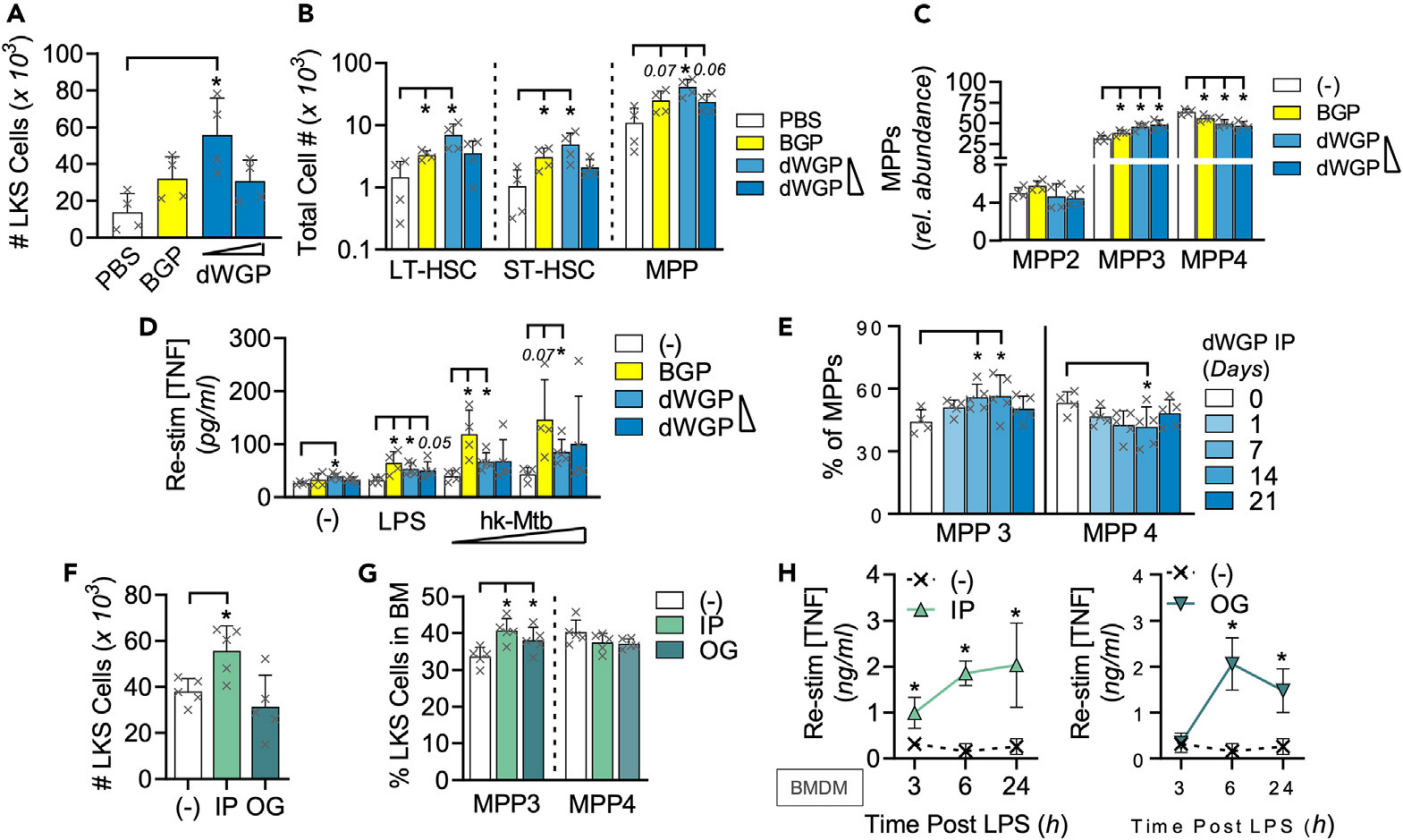
dWGP delivery drives myeloid bone marrow reprogramming (A–C) Bone marrow cLin^−^, c-kit^+^, Sca-1^+^ (LKS) populations in C57/BL6J mice 7-day post intraperitoneal (IP) injection of dWGP (0.2 & 0.4 mg/mouse), BGP (1 mg/mouse) or vehicle PBS. Plots show total LKS numbers per femur (A), long-term hematopoietic stem cells (LT-HSC), short-term HSCs (ST-HSC) and multipotent progenitors 1–3 (MPP) (B), or specific subsets within the MPP compartment (C). (D) TNF production 6 h after LPS stimulation (10 ng/mL, 6 h) or heat-killed *Mycobacterium tuberculosis* H37Ra treatment (hk-Mtb, 500–1000 μg/mL) in splenic macrophages from mice in A. (E) MPP3/MPP4 subset frequency in bone-marrow from C57/BL6J mice after IP injection with 0.2 mg dWGP for 1–21 days or PBS vehicle. (F–H) LKS populations (Total LKS; F or MPP3/4 subset frequency; (G) or TNF production in LPS (1 ng/mL) stimulated BMDM (H) from bone marrow taken from C57/BL6J 7-day post-delivery of 0.2 mg dWGP by oral gavage (OG) or IP injection or given PBS via OG. All data are mean ± SEM for n = 4–5 mice per group. ^∗/#^p < 0.05, or indicated p ≥ 0.05 determined using multiple comparisons testing following following one or two-way ANOVA. See also Figure S7.

We found that IP injection of dWGP led to an increase in total bone-marrow LKS+ HSPC cells 1 week after administration (Figures 7A and S7A), in a similar fashion to that previously observed with BGP injection.^13^ This increase was stronger with lower amounts of dWGP β-glucan. There was significant expansion in the absolute numbers of long-term (LT) and short-term (ST) HSPCs (Figure 7B), indicative of increased metabolic and proliferative activity required to supply HSPC turnover, although the overall frequency of each subset was not significantly altered with dWGP (Figure S7B). However, looking within the more abundant lineage-committed MPP cells, we observed a marked increase in the relative abundance of myeloid-committed MPP3 cells, at the expense of lymphoid-progenitor MPP4 in mice injected with both concentrations of dWGP employed, as observed with an equivalent high concentration of BGP (Figure 7C). The abundance of megakaryocyte-linked progenitors MPP2 was not significantly altered across treatments. As well as increased myelopoiesis, training stimuli have been shown to reprogram HSPCs for enhanced activity upon maturation,^43^ consistent with what has been observed for peripheral monocyte training. Splenic macrophages were isolated and stimulated with LPS or various concentrations of heat-killed Mtb (hk-Mtb). Splenic macrophages from animals trained *in vivo* with BGP or the lower concentration of dWGP displayed significantly enhanced TNF production relative to splenocytes from control injected mice (Figure 7D). These data indicate that IP delivery of particulate β-glucans can lead to mouse bone marrow reprogramming, quantitatively increasing myeloid cell turnover and qualitatively altering their phenotype such that resulting mature cells have a heightened response to activation.

A major question regarding trained immunity remains the longevity of innate memory effects. Therefore, we conducted a time-course analysis of the effects on bone marrow myelopoiesis after IP injection of WGP. Consistent with earlier results, when C57/BL6J mice were injected with 0.2 mg dWGP, we found a significant increase in the percentage of MPP3 cells, at the expense of MPP4 cells, 7 days post-injection (Figure 7E). This was not seen 1-day after dWGP injection and was maintained up to 14 days post-injection, but began to reduce by 28 days. These results are consistent with earlier studies of β-glucan injection which demonstrated a dynamic remodeling of bone marrow progenitors over time.^13^

### Oral delivery of dWGP reprograms bone marrow macrophage responses

β-Glucans represent a key class of non-digestible dietary fiber present in many foods and supplements.^14^^,^^44^ Yeast β-glucans in particular are well tolerated and safe.^45^ We therefore wondered if oral delivery of dWGP could drive training effects *in vivo*. First, we delivered a dose equivalent to the amount injected IP (0.2 mg dWGP) but via oral gavage (OG). Bone marrow was taken 1-week post dWGP delivery and examined for HSPC subsets. Although an expansion of total LKS+ numbers similar to that observed with IP delivery was not observed by OG administration (Figure 7F), the ratio of myeloid-committed MPP3 cells did increase in mice given dWGP by both IP and OG routes (Figure 7G). Splenocytes from OG-treated mice but not IP-treated mice demonstrated significantly enhanced TNF production after LPS treatment (Figure S7C). While BMDMs generated from all treated mice showed enhanced kinetics in TNF production after low-dose stimulation with LPS (Figure 7H). These qualitative and quantitative differences in bone marrow effects are likely as a result of different routes of administration of training agents. However, these data support the notion that oral administration of β-glucans can enhance innate immune functions.

### β-glucan-containing diets drive features of trained immunity

**Figure 8:**
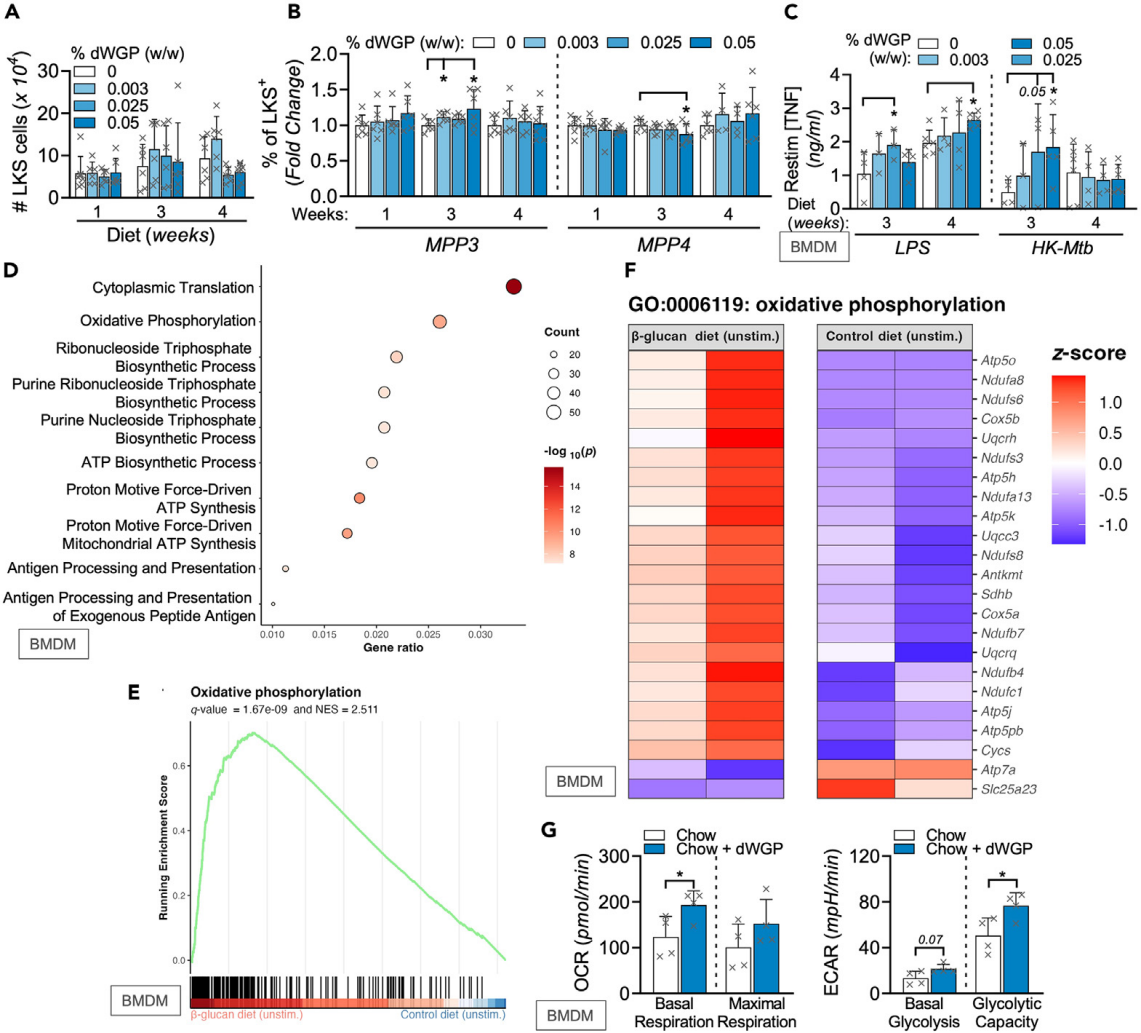
dWGP-containing diets reprogram bone marrow macrophage responses (A and B) Bone marrow cLin^−^, c-Kit^+^, Sca-1^+^ (LKS) populations in C57/BL6J mice fed a control (0% dWGP diet) for 2 weeks prior to initiation of diets supplemented with increasing concentrations of dWGP (0, 0.003%, 0.025%, 0.050% per kg chow) for the indicated weeks. Plots show total LKS cells per femur (A) or MPP3/4 subset frequency (B). (C) TNF production in BMDMs from mice fed WGP-containing diets for 3–4 weeks after stimulation with LPS (1 ng/mL) or hk-Mtb, (500 μg/mL) for 24 h. (D and E) RNA-sequencing analysis of BMDMs generated from mice fed 0.025% WGP diet for 1 week. Plots show GO enrichment (D), gene set enrichment for oxidative phosphorylation (E), and expression of associated genes (F) between unstimulated BMDMs from control or β-glucans WGP-fed mice. (G) Extracellular flux analysis in BMDMs derived from mice fed a dWGP-enriched diet (0.05%, Chow + dWGP) or control chow for 4 weeks. Basal and maximal glycolytic and respiration rates were calculated as before. Data are mean ± SD for n = 6–8 (A–C) or n = 2 (D, E) or n = 3 (G) mice per group. ∗p < 0.05 or indicated p ≥ 0.05 determined using multiple comparisons testing following mixed effect model (A–C) or Student’s t tests (D–G). Log_10_ p values, *Z* scores and enrichment scores for RNA-sequencing are indicated on plots. See also Figure S8.

To examine this in a more relevant setting, we designed a feeding study whereby increasing doses of dWGP, incorporated as the commercially available ingredient Wellmune,^46^ were fed to groups of mice alongside control diets enriched equivalently with an inert non-digestible dietary fiber (inulin) to match for dietary energy and fiber intake for up to 4 weeks. Similar to our OG study, we did not observe an expansion in total bone marrow LKS+ cell numbers in dWGP-fed mice (Figure 8A). Despite this, dynamic changes were observed in frequencies of the LKS+ populations overtime, with a decrease in LT-HSC frequency in mice after 3 weeks of feeding and no significant impact on ST-HSCs (Figures S8A and S8B). Importantly, the levels of the more abundant MPPs were enhanced after 3-week dWGP feeding (Figure S8C). Within these, the ratio of myeloid-committed MPP3 was significantly increased in mice fed higher concentrations of dWGP-containing diets relative to control chow at 3-week post-feeding, with a significant decrease in levels of MPP4 cells (Figure 8B). Importantly, mature BMDMs derived from these mice displayed increased sensitization to stimulation. BMDMs generated from mice at later times (3–4 weeks) post-feeding displayed enhanced TNF production in response to stimulation with low concentrations of LPS or hk-Mtb (Figure 8C). RNA-seq analysis was then performed on BMDMs taken from dWGP-fed mice. No significant differences in gene expression were observed in LPS-treated BMDMs from dWGP-fed mice (data not shown). However, when basal differences in gene expression were compared in unstimulated BMDMs, more significant changes were detected (Figures S8D). Gene enrichment analysis revealed that many of these genes were involved in the processes of protein translation, antigen presentation, and cellular metabolism, including oxidative phosphorylation (Figures 8D–8F).

To confirm the functional consequences of our *in vitro* and RNA-seq analysis that suggested metabolic adaptions in trained cells, BMDMs were taken from mice fed a modified diet supplemented with dWGP as Wellmune (Chow-dWGP) or a standard mouse chow diet for 4 weeks. Examining metabolic flux, we observed a significant enhancement in basal OCR levels in BMDMs derived from dWGP-fed mice (Figure 8G). Although basal glycolysis is not significantly enhanced (p = 0.07), when interrogated with inhibitors, glycolytic capacity is significantly enhanced in BMDMs from dWGP-fed mice. This confirms that oral supplementation with dWGP leads to functional reprogramming of myeloid progenitors with enhanced immunometabolic responses in mature progeny. In conclusion, dWGP is a yeast-derived β-glucan particle which can drive trained responses in innate immune cells – both macrophages and bone marrow progenitors.

## Discussion

Our investigations uncovered a pathway whereby recognition of intact fungal particles and subsequent internalization via the phagocytic synapse^9^ is intimately linked to metabolic investment by the target cell into a trained phenotype. Although our studies used artificial β-glucan-rich particles to engage Dectin-1 in this way, it is tempting to speculate that this represents a conserved pathway by the innate immune system to avoid inappropriate and wasteful resources in long-term memory responses to soluble ligands or non-viable pathogens and rather to promote a trained phenotype only when necessary. This involves reprogramming cellular metabolism which includes upregulating glycolysis, but also maintaining and upregulating oxidative metabolism. Importantly, these changes require internalization of β-glucan/Dectin-1 complexes and phagolysosomal activity. This metabolic reprogramming—both glycolysis and TCA-modulation—is linked to the epigenetic changes which underline the trained phenotype and accelerated response to non-specific restimulation.^10^^,^^28^^,^^34^ Having observed this pathway in monocytes which are short-lived unless they migrate to tissues as macrophages,^47^ we also observed the ability of artificial β-glucan-rich particles (dWGP) to affect myeloid progenitor cells in the bone marrow and enhance functional responses in mature macrophages.^41^ Thus, exploiting the physical and chemical requirements for optimal trained responses could be used to improve innate immune function for therapeutic approaches like vaccination or immunotherapies, or to promote increased resistance against novel pathogens to which we have no pre-existing acquired memory, like the recent scourge of COVID-19 or its variants.^12^^,^^14^^,^^48^^,^^49^ Intriguingly, in the post-COVID world where long-term immune dysregulation has been described,^50^ we hypothesize that β-glucan training to generate a balanced innate response could protect against increased severity to other infections.

Our study highlights that it is not just β-glucans from pathogenic fungal strains like *C. albicans* which can promote trained immunity,^4^^,^^11^ but common yeast β-glucans possess that capacity too. Importantly, the way β-glucans interact with the immune system is key to dictating the outcome of this response.^9^^,^^27^ Dectin-1 recognizes the characteristic chemical β-1,3 and β-1,6 linkages between glucose units to distinguish fungi from other more inert β-glucans like oat and algal sources and thereby drive characteristic pro-inflammatory responses like NF-κB and pro-inflammatory cytokine production in acute innate responses.^6^^,^^7^ However, it is the physical recognition by the same receptor and the subsequent activation of the phagocytic machinery,^9^ which commits the cell to a trained response. Consistent with our screening studies and recent observations made by multiple other groups,^4^^,^^11^^,^^26^^,^^34^^,^^51^ an array of diverse β-glucans can drive trained immunity. However, the exact mechanism whereby these specific ligands drive the metabolic reprogramming required for training has not been delineated. It may be that these diverse β-glucan preparations also contain high-MW chains or particulates which drive the phagocytic synapse or alternatively possess other undefined characteristics unique to each, not shared with dWGP. Indeed, dWGP itself represents an artificial β-glucan preparation, rich in β-glucan which has had contaminating mannan and other TLR2 ligands removed.^24^ It has been observed in *C. albicans* that outer cell wall mannans block β-glucan interacting with the innate immune system and driving phagocytosis^52^ and thus mannans may have emerged as an immune evasion strategy to block training of the host by β-glucan exposure. This may explain why in our hands, ZYM, which represents a particulate *S. cerevisiae* cell wall preparation containing β-glucans masked by a mannan layer,^24^ was unable to drive the same level of trained immunity *in vitro* and instead promotes acute inflammatory responses. Removal of these mannans conferred the capacity to metabolically reprogram and train cells with depleted ZYM preparations. It remains to be seen whether unmasked dWGP itself mimics the natural state of an *S. cerevisiae* infection either in budding yeast or hyphal form. The finding that larger WGP-aggregates lack the ability to drive Dectin-1 internalization and metabolic reprogramming associated with training suggests that this may represent a response to viable, budding yeast specifically. More filamentous hyphal species like *Aspergillus fumigatus* are known to evade immunostimulatory responses by altering β-glucan content and physically masking it.^53^^,^^54^ Therefore, the finding that WGP and other β-glucan preparations represent strong drivers of trained immunity may in fact be an artificial observation that does not occur in the wild, but which we can exploit to promote immune function.

Regardless of whether this represents a natural response to yeast infection, this work reveals the importance of mode of presentation of β-glucan chains which determines their solubility^24^ and ultimately, their interaction with the innate immune system.^27^ Soluble yeast β-glucans have been employed in both immunotherapeutic approaches and nutritional supplementation strategies for some time.^14^^,^^55^^,^^56^ Although our data suggest solubilized WGP cannot train and instead blocks receptor occupancy by higher MW particulate β-glucans, other soluble β-glucans can drive trained responses both *in vitro* and *in vivo*, including *T. versicolor* BGP (Figures 1 and 6 and ref. 13). Here, we employed BGP as a positive control in our assays^26^ and found that it drives many of the same features as dWGP, including phagolysosomal maturation and upregulation of ox-phos. This may seem surprising given its solubility in water. However, because of its highly rhamnified nature, conferred by the unique peptide linkages connecting glucan chains,^57^ we hypothesize that it engages the Dectin-1 phagocytic synapse. In doing so, it causes the characteristic Dectin-1 receptor clustering required for phagocytosis, albeit to a less efficient extent as particulate β-glucans like dWGP and ZYM-d. While particulate β-glucans trigger trained immunity, other soluble β-glucans sWGP included,^26^ could have immune-promoting or training effects through other receptors or cellular substrates—including binding CR3 in neutrophil priming^58^^,^^59^ or by tempering excessive Dectin-1 activation by particulate β-glucans.^9^^,^^27^ Similarly, lower MW chitin fragments have been shown to trigger different responses than larger fragments.^60^ While intact preparations of this common fungal cell wall carbohydrate are largely immunologically inert, intermediate fragments preferentially trigger pro-inflammatory cytokines via TLR2. Shorter chitin fragments engage Dectin-1 to drive anti-inflammatory responses like IL-10, in a process regulated by phagocytic receptors. Although our lower MW sWGP β-glucan preparations gained the ability to bind Dectin-1b, they did not drive trained immunity, highlighting that receptor interaction itself is not sufficient for this process. Instead, internalization via phagocytosis emerges as a key step regulating long-term responses.

Both BGP, dWGP and ZYM-d maintain and upregulate ox-phos unlike other β-glucan preparations which led to tolerance. This finding builds on observations by Domínguez-Andres et al. which places disruptions of TCA cycling, via IRG1 activity, as key to dictating tolerance or training phenotypes.^20^ Initial work on *C. albican*s β-glucan training in human cells implicated glycolytic reprogramming as a key metabolic event required for reprogramming.^10^^,^^28^ While our data demonstrate this is an important event downstream of β-glucan recognition, it does not completely distinguish training capacity. Instead, our observations, alongside emerging data using higher concentrations of *C. albican*s β-glucan in human cells^37^ or β-glucan training in mouse cells^15^^,^^18^ implicate upregulation of ox-phos and anaplerotic feeding of TCA as crucial molecular events for a training phenotype. Although this is likely linked to the epigenetic modifications required for trained responses,^34^^,^^37^ it could also be connected to other processes linked to enhanced responsiveness. Enhanced proliferation has been shown in training models with *C. albicans* (β-1,3)-glucans,^11^^,^^28^ and enhanced growth factor signaling was recently linked to differentiation and training for other yeast β-glucan preparations.^51^ Since upregulation of ox-phos is a feature of more long-lived lymphocytes and reparative macrophages,^61^ remodeling of TCA and up-regulation of oxidative metabolism could emerge as central for long-term survival and memory responses in both innate and adaptive cells.

In conclusion, our work herein has identified an immunomodulatory role for yeast-derived β-glucan particles through driving metabolic reprogramming in target cells required for trained immunity. This may affect strategies using these and similar β-glucans to promote innate immune function and may be useful in promoting innate immune resistance to infection. At the same time, our findings imply that the pathway of driving trained immunity may have evolved as a bioenergetic response to perceived threats by primitive pattern recognition receptors, such that the investment of metabolic and energetic resources in long-term innate memory-like responses is only committed upon specific recognition of valid infectious threats. Although pathogens may have evolved to counteract this in their modes of ligand presentation, further analysis of these characteristics across the range of molecules now described to drive trained immunity in other contexts could point to superior ways to modulate this response for improved therapeutic benefit.

### Limitations of the study

Although the biological significance of *S. cerevisiae*-derived β-glucans driving trained immunity is discussed previously, our findings also have implications for the application of β-glucans in therapeutic approaches. As particles, their utility in immunotherapeutics is limited. The dWGP employed here, sold as Wellmune, has been studied for its effect on upper respiratory tract infections in elite marathon runners, as well as altering circulating cytokine levels and LPS-induced monocyte production in immunocompromised populations after oral consumption.^46^^,^^55^^,^^62^^,^^63^ Our finding that oral delivery of dWGP has biological and training-like effects in mice opens up avenues for exploitation of this class of molecule commercially and therapeutically. Notably, the effects on mouse bone marrow myelopoiesis through the 2 distinct routes examined were distinct. IP injection of dWGP is known to drive an inflammatory response^64^ and here we show this impacts bone marrow hematopoiesis by both quantitively increasing HSPC numbers and qualitatively skewing population frequencies toward myelopoiesis. Oral delivery, either through gavage or dietary incorporation, does not drive such a dramatic bone marrow expansion, but does skew toward myelopoiesis and increases the sensitivity of mature progeny. Therefore, whether sensing of particulate β-glucans in the gut^65^ can transmit a signal to alter bone marrow hematopoiesis requires further examination. In particular, the effect of beta-glucans on both the resident mucosal immune cells or the endogenous gut microbiome could impact long-term innate memory *in vivo*. Equally, continual exposure to β-glucan in the diet may also explain the minimal long-term effects observed at the transcriptomic level, which mimics previous observation in humans fed a similar β-glucan for 1 week.^66^ Further studies will delineate the temporal requirement and lifespan of both bone marrow expansion and myeloid reprogramming through pulse-chase delivery of oral β-glucans.

## STAR★Methods

### Key resources table

**Table:** 

REAGENT or RESOURCE	SOURCE	IDENTIFIER
**Antibodies**		

anti-human Dectin-1-PE	Biolegend	clone 15E2
anti-human CD14-APC	Biolegend	clone M5E2
anti-human CD16-PE-Cy7	Biolegend	clone 3G8
anti-human HLA-DR-BB515	BD Bioscience	clone G46-6
anti-human phospho-S6-PE (Ser235/236)	Cell Signalling	clone D57.2.2E
anti-mouse CD11b–APC	Biolegend	clone M1/70
anti-mouse F4/80–PeCy7	Biolegend	clone BM8
anti-mouse TNF–BV421	Biolegend	clone MP6-XT22
anti-mouse CD16/32	Biolegend	clone 93
anti-mouse Ter-119-Biotin	Biolegend	clone TER-119
anti-mouse CD11b-Biotin	Biolegend	clone M1/70
anti-mouse CD5-Biotin	Biolegend	clone 53-7.3
anti-mouse CD4-Biotin	Biolegend	clone RM4-5
anti-mouse CD8a-Biotin	Biolegend	clone 53-6.7
anti-mouse CD45R+-Biotin	Biolegend	clone RA3-6B3
anti-mouse c-Kit-APC	Biolegend	clone 2B8
anti-mouse Sca-1PE-Cy7	eBioscience	clone D7
anti-mouse CD150-eFluor450	eBioscience	clone mShad1
anti-mouse CD34-FITC	eBioscience	clone RAM34
anti-mouse Flt3-PE	Biolegend	clone A2F10.1
Streptavidin-APC-Cy7	Biolegend	# 405208

**Bacterial and virus strains**		

*Mycobacterium tuberculosis* H37Ra	ATCC	25177

**Biological samples**		

Buffy packs from human blood donations	Irish Blood Transfusion Service, St James’ Hospital, Dublin	N/A

**Chemicals, peptides, and recombinant proteins**		

dWGP – dispersible whole-glucan particle derived from *Saccharomyces cerevisiae*	Kerry Group	N/A
sWGP - solubilized whole-glucan particle derived from *Saccharomyces cerevisiae*	Kerry Group	N/A
sWGPs F1-F4 – molecular weight fractions of solubilized whole-glucan particle derived from *Saccharomyces cerevisiae*	Kerry Group	N/A
BGP – Beta glucan peptide derived from *Trametes versicolor*	Invivogen	tlrl-bgp
Zymosan – crude Beta glucan particle derived from *Saccharomyces cerevisiae*	Invivogen	tlrl-zyn
Depleted Zymosan – pure Beta glucan particle derived from *Saccharomyces cerevisiae*	Invivogen	tlrl-dzn
Schizophyllan - Beta glucan preparation derived from *Schizophyllum commune*	Invivogen	tlrl-spg
Curdlan - Beta glucan preparation derived from *Alcaligenes faecalis*	Invivogen	tlrl-cura
Laminarin - Beta glucan preparation derived from *Laminaria digitita*	Invivogen	tlrl-lam
Heat-killed *Candida albicans*	Invivogen	tlrl-hkca
Heat-killed *Mycobacterium tuberculosis* H37Ra	Invivogen	tlrl-hkmt-5
Irradiated *Mycobacterium tuberculosis* H37Rv	BEI Resources	NR-14819
Ultrapure lipopolysaccharide from *E.coli* O111:B4	Invivogen	tlrl-3pelps
PAM3CSK4, synthetic triacylated lipopeptide	Invivogen	tlrl-pms
Poly(I:C), high molecular weight polyinosine-polycytidylic acid	Invivogen	tlrl-pic
5’methylthioadenosine (MTA)	Sigma-Aldrich	M1949
Piceathanol (PIC)	Sigma-Aldrich	P0453
BAY 11-7082	Cayman Chemicals	10010266
Cytochalasin-D	Sigma-Aldrich	C8273
2 deoxyglucose	Sigma-Aldrich	D6134
Oligomycin	Sigma-Aldrich	495455
Rapamycin	Sigma-Aldrich	553210
Wortmannin	Thermo-Fisher	PHZ1301
di-methyl-malonate	Sigma-Aldrich	136441
GLS1 Inhibitor III, CB839	Sigma-Aldrich	5.33717
Bafilomycin-A1 from *Streptomyces griseus*	Sigma-Aldrich	B1793
fluoro-carbonyl cyanide phenylhdrazone (FCCP)	Sigma-Aldrich	C2920
rotenone	Sigma-Aldrich	R8875
Antimycin A from *Streptomyces* sp.	Sigma-Aldrich	A8674
Zombie Aqua™ Fixable Viability Kit	Biolegend	423102
Recombinant Human M-CSF	Peprotech	300-25
3 μm aminated polystyrene particles	Magsphere	PS003UM
1,1′-Carbonyldiimidazole (CDI)	Sigma-Aldrich	21860

**Critical commercial assays**		

Human TNF Uncoated ELISA Kit	Invitrogen	Cat #88-7346-88
Mouse TNF Uncoated ELISA Kit	Invitrogen	Cat #88-7324-88
Human IL-8 (CXCL8) Uncoated ELISA Kit	Invitrogen	Cat #88-8086-88
Human IL10 Uncoated ELISA Kit	Invitrogen	Cat #88-7106-88
Mouse IL10 Uncoated ELISA Kit	Invitrogen	Cat #88-7105-88
Mouse IL-6 Uncoated ELISA Kit	Invitrogen	Cat #88-7064-88
Mouse IL-1b Uncoated ELISA Kit	Invitrogen	Cat #88-7013A-88
Mouse IL-2 DuoSet ELISA	R&D Systems	DY402
Seahorse XFe96/XF Pro Cell Culture Microplates	Agilent	103794-100
Seahorse XF DMEM assay medium pack, pH 7.4	Agilent	103680-100
QuantiBlue HEK-Blue™ Detection reagent	Invitrogen	hb-det3
Lactate Assay Kit	Sigma-Aldrich	MAK064
MitoTracker™ Green FM (MTG)	Thermo-Fisher	M7514
Image-iT™ Tetramethylrhodamine, methyl ester (TMRM)	Thermo-Fisher	I34361
UltraComp eBeads™ Compensation Beads	Invitrogen	01-2222-42

**Deposited data**		

Raw data and analysis Excel files, deposited in Mendeley Data, V1,	*This paper*	Medeley Data: https://doi.org/10.17632/kfzjtrmthb.1
Bulk RNA-Seq of BMDM from WGP-fed mice	GEO	GEO: GSE235691
R-code used for RNA-Seq analysis	*This paper*	GitHub

**Experimental models: Cell lines**		

HEK-Blue hDectin1a	Invivogen	hkb-hdect1a
HEK-Blue hDectin1b	Invivogen	hkb-hdect1b
HEK-Blue hTLR2	Invivogen	hkb-htlr2
HEK-Blue hTLR4	Invivogen	hkb-htlr4
RAW-Blue	Invivogen	raw-sp

**Experimental models: Organisms/strains**		

C57/BL6J	Comparative Medicine Unit, Trinity College Dublin	*in-house colony*

**Oligonucleotides**		

Citrate synthase (mouse) Taq Man primer/probes	Thermo Fisher	Mm00466043_m1
Fumarate hydratase 1 (mouse) Taq Man primer/probes	Thermo Fisher	Mm01321349_m1
Hexokinase 2 (mouse) Taq Man primer/probes	Thermo Fisher	Mm00443385_m1
HPRT (mouse) Taq Man primer/probes	Thermo Fisher	Mm01545399_m1
Lactate dehydrogenase-A (mouse) Taq Man primer/probes	Thermo Fisher	Mm01612132_g1
Malate dehydrogenase-2 (mouse) Taq Man primer/probes (mouse)	Thermo Fisher	Mm00725890_s1
Phosphofructokinase-M isoform (mouse) Taq Man primer/probes	Thermo Fisher	Mm01309576_m1
Slc2a1 (mouse) Taq Man primer/probes	Thermo Fisher	Mm00441480_m1
Suclg1 (mouse) Taq Man primer/probes	Thermo Fisher	Mm00451244_m1

**Software and algorithms**		

Microsoft Office Excel	Microsoft	https://www.microsoft.com
Microsoft Office Powerpoint	Microsoft	https://www.microsoft.com/
ImageStream Data Analysis and Exploration Software (IDEAS)	Luminex	V6.3
FACS Diva	BD LifeSciences	v9.0
FlowJo	BD LifeSciences	v10.8
Prism v9.3.0	GraphPad	https://www.graphpad.com/
R studio	R Studio 2020 Team	http://www.rstudio.com/

**Other**		

Standard mouse chow diet, used for modified diets (below)	Research Diets	D11112201
Mouse diets with additional inulin:Wellmune (dWGP) as described in Methods	Research Diets	*This study*

### Resource availability

#### Lead contact

Further information and requests for resources and reagents should be directed to the lead contact, Prof. Frederick J Sheedy (fsheedy@tcd.ie).

#### Materials availability

This study did not generate new unique reagents.

#### Data and code availability

• RNA Sequencing data is deposited and publically available at the Gene Expression Omnibus (GEO) with the accession number GSE235691, as listed in the key resources table. All data and statistical analysis reported in this paper is available in Mendeley Data under https://doi.org/10.17632/kfzjtrmthb.1.
• Code used for RNA-Seq analysis is available on GitHub at https://github.com/aaron-breathnach/wgp_rna_seq.
• Any additional information required to reanalyse the data reported in this paper is available from the lead contact upon request.

### Experimental model and study participant details

#### Animals

C57BL/6J male mice were generated and maintained at the Comparative Medicine Unit, Trinity College Dublin (Dublin, Ireland). Mice were bred and maintained under specific pathogen-free conditions with *ad libitum* access to food and water. Mice were used at the age of 8-12 weeks. All experiments were carried out under the approval of the Health Products Regulatory Authority, Ireland and Trinity College Dublin Animal Research Ethics Committee.

#### Human material

Human peripheral blood mononuclear cells (PBMCs) were isolated from anonymously donated buffy coats obtained from the Irish Blood Transfusion Services (Dublin, Ireland). Supply of human blood products from IBTS was approved by clinical indemnity and approved by Trinity College Faculty of STEM Level 1 Research Ethics Committee. Information on biological sex and age of participants was not provided.

#### Cell lines

HEK293-Blue and RAW-Blue NFκB-SEAP reporter cells were obtained from Invivogen, including hDectin1b, hDectin1a, hTLR2 and hTLR4 overexpressing HEK-Blues. All HEK reporter cell lines were derived from human embryonic kidney cells and are female. Each cell line was authenticated by testing with standard positive and negative control stimuli. Cell lines tested negative for mycoplasma contamination.

### Method details

#### Cell isolation

Bone marrow derived macrophages (BMDM) were isolated by flushing the tibia and femur from both legs of C57BL/6J mice with DMEM (Gibco). The suspension of bone marrow cells obtained was strained on a 40μM nylon mesh cell retainer (Biolegend). After a wash, the pellet was resuspended and treated 2 minutes with Red Blood Cell (RBC) Lysis Buffer Hybri-Max™ (Sigma-Aldrich) to lyse erythrocytes. After washing and counting, cells were resuspended in DMEM, 10% FBS, 20% L929-conditioned media and seeded to be differentiated into BMDMs over 1 week. Mature BMDM were lifted in cold PBS and cells reseeded at the required density in DMEM, 10% FBS, 5% L929-conditioned media and allowed to rest overnight before stimulation. Human PBMCs were isolated from human blood-derived buffy packs using density gradient centrifugation with Lymphoprep (Stem Cell Technologies) followed by Red Blood Cell lysis using Lysis Buffer Hybri-Max™ (Sigma-Aldrich). PBMCs were resuspended in differentiation media (cRMPI, 10% FBS, 50 ng/mL M-CSF) and monocytes enriched by adherence to plastic and used for subsequent training assays or stimulations.

#### β-glucan preparations and training stimuli

*Saccharomyces cerevisiae*-derived whole glucan particles were provided by Dr Sonja Nodland, Kerry Health & Nutrition, Minnesota, USA. These include dispersible whole-glucan particles (dWGP) in which cell wall β-glucan was preserved – yielding ghost yeast cells – or a soluble preparation, derived from heat-treated dWGP. We have referred to this formulation as soluble WGP (sWGP) to highlight that dWGP and sWGP are composed of β-glucans from the same source. Dr Nodland also provided β-glucan fractions of differing molecular weights that were isolated from sWGP by size exclusion chromatography, followed by filtration (1.5-100 nm filtration steps). The fractions were as follows: F1: <100 kDa, F2: 100-400 kDa, F3: 400-800 kDa and F4: >800 kDa. For all experiments, bulk WGP powder was resuspended in PBS and sonicated to declump and obtain dispersible “single-cell” ghost particles (dWGP).

The sonication procedure was as follows: bulk WGP was weighed and dissolved in sterile endotoxin-free water (Invitrogen) to yield 10-15 mL of 25 mg/mL WGP. This solution was left at room temperature overnight (8-16 h) before sonication with a 150VT ultra sonic homogenizer with a 5/32” microtip. The solution was sonicated for 5 min, at 50% power and 50% time pulse rate, while the tip was immersed roughly 5 mm below the surface of the liquid. Due to the heat caused by the sonication, the tube containing the solution was kept in ice. Following this sonication step, the dWGP was pelleted via centrifugation (1,000 G, 10 mins, at room temperature) and the water was removed by careful decanting and replaced with 0.2 M NaOH in water at a volume to reach 25 mg/mL WGP. After 20 minutes, the dWGP was washed three times with sterile water, using the same pelleting, decanting and replacement of solvent conditions as described. Finally, two last washes were carried out to replace the sterile water with sterile PBS and stored at 4°C for up to 6 months. As dWGP settles out of solution, it also required vortexing prior to each use. Unsonicated whole-glucan particles (uWGP) were used in some experiments and were resuspended directly in PBS without sonicating.

For some experiments, 3 μm aminated polystyrene particles (AM-PS; Magsphere) were conjugated with sWGP, based on a published method.^67^ 2 mg of AM-PS were washed with anhydrous DMSO (Sigma) three times, using centrifugal filters containing 0.65 μm PVDF membrane (Ultrafree, Millipore) before incubation with 250 μL of 2 M 1,1′-Carbonyldiimidazole (CDI; Sigma), freshly dissolved in anhydrous DMSO, for one hour at room temperature, whilst rocking. The particles were then washed twice with anhydrous DMSO to remove excess CDI (using centrifugal filters), prior to incubation with 250 μL of 0.1 mg/mL sWGP, dissolved in anhydrous DMSO, for one hour at room temperature, whilst rocking. Following this conjugation step, the sWGP was collected by centrifugation, using centrifugal filters to block the particles, and the efficiency of conjugation was assessed by measuring the loss of sWGP, using filtered sWGP solutions without AM-PS, or CDI, as controls. The sWGP was measured using a phenol sulphuric acid method, based on a reported protocol for carbohydrates.^68^

Other β-glucans were obtained from Invivogen and include; Beta-glucan peptide (BGP) a high molecular weight polysaccharide extracted from the macrofungus *Trametes versicolor*, Zymosan, a *Saccharomyces cerevisiae*-derived cell wall preparation, Schizophyllan, a high molecular weight β-glucan derived from the fungus *Schizophyllum commune*, Curdlan, a β-1,3 linked glucan derived from the bacteria *Alcaligenes faecalis*, Pustulan, a median molecular weight linear β-1,6 linked glucan from the algal lichen *Lasallia pustulata*, Laminarin, a low molecular weight soluble β-1,3, β-1,6-glucan from *Laminaria digitata*, Zymosan, a particulate *Sacchorymces cerevisiae* β-glucan rich cell wall preparation and Zymosan-depleted, a form of Zymosan treated with hot alkali to remove TLR-stimulating properties. Further information is provided in Table 1. All β-glucans were used at concentrations between 1, 10 or 100 μg/mL. Heat-killed Candida albicans (HKCA) was also obtained from Invivogen and used at concentrations between 1x10^4-6^ cells/mL.

#### Training assays & readouts

For human monocyte Training Assays, PBMCs isolated from human blood were seeded in 96 well plates (100,000 cells per well in 180 μL RMPI, 10% FBS, 50 ng/mL M-CSF) and 20 μL of the training stimulus was added immediately. Cells were incubated overnight at 37°C. Media was removed and cells were very gently washed twice with 100 μL of warm PBS to remove training stimulus and 200 μL of fresh media was added. Cells were washed and given fresh media every 2-3 days and 5-days post isolation cells were re-stimulated in fresh media for the indicated times. BMDM Training Assays were performed by stimulating mature BMDM with training stimuli 6-day post-isolation and allowing to recover and mature for a further 6-days in DMEM, 10% FBS, 5-7% L929-conditioned media, changing the media every 3 days prior to restimulation. For training inhibition assays, PBMC cells or BMDM were incubated with the desired inhibitor for the indicated time prior to the addition of the training stimulus. Inhibitor concentrations were as follows unless specifically indicated: 1 mM 5’methylthioadenosine, an excess of sWGP (10-100 μg/mL), 10-100 μg/mL laminarin (Invivogen), piceatannol between 4, 10 and 30 μM, 5 μM BAY 11-7082, between 1, 2.5, 5 and 10 mM 2-deoxyglucose, between 1, 10 and 100 nM Rapamycin, between 0.1, 1 and 10 μM wortmannin, between 2, 10 and 100 μM Cytochalasin D, between 1, 5 and 10 μM bafilomycin A1, Oligomycin (20 μM), di-methy-malonate (DMM, 10 mM) & CB-839 (1 μM). All inhibitors were from Sigma-Aldrich unless otherwise indicated. As a readout of training, TNF secretion from trained cells was measured by ELISA of cell-free supernatants (human or murine TNF ELISA kits Invitrogen). Alternatively, human CXCL8 or IL-10 production or mouse IL6 and IL-10 production was measured by ELISA (Invitrogen). Media removed from BMDM after 24-72h treatment was analysed for cytokine production using ELISA. Mouse IL-1β and Mouse IL-2 were also measured (R&D Biosystems). For supernatant transfer experiments, supernatants from human monocytes 24h post-training were harvested and administered to naïve, untrained monocytes alongside dWGP-trained monocytes, matured for 5-days prior to restimulation with LPS to assess if soluble factors induced by training stimuli conferred enhanced responsiveness to restimulation.

#### Macrophage activation/Restimulations

Ultrapure lipopolysaccharide (LPS) from *E.coli* O111:B4 was obtained from Invivogen and used to induce tolerance in human monocytes or BMDM at concentrations of 1, 10 and 100 ng/mL or used to restimulate trained macrophages at 10 ng/mL in most experiments or between 1 and 100 ng/mL. Pam3CSK4, a synthetic triacylated lipopeptide TLR2/TLR1 agonist was obtained from Invivogen and used for restimulation at 10 μg/mL. Poly(I:C), a dsRNA mimetic and TLR3 agonist was used at 5 μg/mL (Invivogen). HKCA was used to restimulate trained macrophages at 1x10^6^ cell/mL or Zymosan was used as above. Trained monocyte-derived macrophages were restimulated with non-viable irradiated *Mycobacterium tuberculosis* (iMtb) obtained from the BEI Resources (ATCC) and prepared according to manufacturer’s instructions and used at 500 μg/mL. BMDM from *in vivo* trained mice were stimulated with heat-killed *Mycobacterium tuberculosis* (hk-Mtb) from Invivogen and used at concentrations between 500 and 1000 μg/mL. Trained monocyte-derived macrophages were also infected with viable *Mycobacterium tuberculosis* (Mtb) strain H37Ra obtained from ATCC and propagated in Middlebrook 7H9 medium to log phase. On the day of infection, bacteria in log-phase were pelleted by centrifugation and resuspended in DMEM. Bacterial pellets were de-clumped by passing through a syringe with an 25G needle several times. A single cell suspension was isolated by centrifuging the bacterial suspension at 800 rpm for 3 min. The supernatant of this spin was quantified by spectrophotometry (OD_600nm_) and used to infect macrophages. Macrophages were infected at an MOI of 5 bacilli per cell for 3h (as described^51^), purified further by centrifugation at 13,000 rpm for 10 min to pellet extracellular bacteria. Bacteria-free media was returned to macrophages after washing in DMEM to remove extracellular bacteria and cultures grown up to 72h post-infection. For experiments where trained macrophages were infected with *Mycobacterium tuberculosis* (Mtb), baseline growth was assessed by lysing 3 h time-point in 0.1% Triton-X for 10 min. Serial dilutions were plated on 7H10 Middlebrook Agar in triplicate and colony-forming units enumerated after incubation at 37°C for 14–21 days after plating. For later growth measurements this lysate was combined with pelleted extracellular bacteria, obtained by centrifugation of supernatant and fold-change in bacterial colony forming units (CFUs) expressed relative to baseline time-point.

#### QuantiBlue assays

HEK-Blue or RAW-Blue NFκB-SEAP reporter cells (Invivogen) were cultured using Puromycin and HEK-Blue™ CLR Selection (Invivogen) as selective antibiotics and DMEM (4.5 g/L glucose), 10% (v/v) fetal bovine serum (FBS), 100 U/ml penicillin, 100 μg/ml streptomycin, 100 μg/ml Normocin and 2 mM L-glutamine. Reporter cell assays were carried out by seeding at 50,000 cells per well in 180 μL in a 96 well flat-bottomed plate and incubating the cells with 20 μL of the indicated agonists overnight. SEAP activity was measured using QUANTI-Blue (Invivogen) according to the manufacturer’s instructions. Briefly, 20 μL of supernatant was added to 180 μL of QUANTI-Blue solution and incubated at 37°C for 15 minutes. Optical density at 620 nm was then measured using a plate reader.

#### Seahorse & metabolic analysis

For metabolic analysis of trained cells, lactate concentration was measured in supernatants using the colorimetric Lactate Assay Kit (MAK064, Sigma-Aldrich). Extracellular flux analyses were carried out using an XFe24 Extracellular Flux analyzer (Seahorse Biosciences) in Seahorse Media freshly supplemented with 10 mM glucose and 2 mM l-glutamine. An adapted version of the XF cell mito-stress test was used to measure key parameters of both mitochondrial and non-mitochondrial function through the oxygen consumption rate (OCR) as well as analysis of the extracellular acidification rate (ECAR) of the media to investigate glycolytic flux. Cells were plated in the seahorse plate at 100,000 cells per well for 24 hour stimulation assays or 50,000 cells per well for 72 hour stimulation assay. Cells were stimulated as described above. For 72 hour measurements cells were washed with PBS and 200uL after 24h of stimulation and fresh cDMEM/5%LCM was added. On the day prior to measurement the calibration cartridge was hydrated with 200 μL of XF Seahorse calibration media and was placed in a non-CO_2_ incubator overnight at 37°C. On the day of the assay, cells were washed X2 with seahorse medium. Each well was then topped up with 180μL of seahorse medium and the plate was placed into a non-CO2 incubator at 37°C for 20 min before the beginning of the Seahorse run. Following calibration and the cell culture plate was loaded for real-time analysis. During the run, the following inhibitors (diluted in seahorse media) were injection to interrogate metabolism oligomycin (2 μM), fluoro-carbonyl cyanide phenylhdrazone (FCCP, 1 μM), rotenone/antimycin-A (0.1 μM/4 μM) and 2DG (30 μM). Normalization for cell number was carried out with a Crystal Violet dye assay.

#### RNA analysis

RNA was isolated from trained BMDM with the PureLink RNA Mini Kit (Thermo-Fisher). For analysis of gene expression, cDNA was prepared with the High-Capacity cDNA Archive kit according to manufacturers’ instructions (Applied Biosystems) and individual mRNAs were monitored with the inventoried mouse TaqMan assays (Applied Biosystems, listed in STAR Methods key resources table). The AB7900HT platform (Applied Biosystems) was used for all PCR, performed in triplicate in FAST mode. Changes in expression were calculated by the change in threshold (ΔΔCT) method with 18S as an endogenous control and were normalized to results obtained in untreated cells. RNA-seq was performed by the sequencing provider Novogene. Libraries were prepared using the NEB Next Ultra RNA Library Prep Kit. Libraries were sequenced using an Illumina NovaSeq 6000. Raw reads were trimmed and quality-filtered using fastp. Processed reads were aligned to the mm10 mouse reference genome using STAR. The number of reads mapping to each gene was counted using HTSeq. Downstream analysis was performed in R. Differential expression analysis was performed using DESeq2, and the resulting *p*-values were adjusted using the Benjamini-Hochberg method. Pathway enrichment analysis was performed using clusterProfiler. Specifically; gene set enrichment analysis (GSEA) was performed using the clusterProfiler::GSEA() function, while gene ontology (GO) enrichment analysis was performed using the clusterProfiler::GSEA() function. For GO enrichment analysis, genes with an adjusted *p*-value < 0.05 were considered to be differentially expressed.

#### Animal work

For *in vivo* induction of trained immunity, immunity was induced in mice with a single intraperitoneal injection of either 200μL of PBS as control or 200μL of WGP resuspended in PBS at 1mg/ml or 2mg/ml. For in-vivo induction of trained immunity by oral supplementation, mice were split into four main experimental groups. The control group was fed a slightly modified standard diet containing 25 g/kg of inulin (2.5% inulin per kg chow) as a source of fiber (Ref. D11112201). The three other groups of mice were fed a diet supplemented with 0.003% (0.03g per kg chow), 0.025% (025g per kg chow) or 0.05% (0.5g per kg chow) of the dietary fiber WGP respectively, including a proportionally reduced amount of inulin to balance the amount of WGP fiber incorporated in the supplemented diets. All groups of mice were allowed two weeks to habituate to the inulin-enriched standard diet before being switched to the WGP-supplemented diets for 1 to 4 weeks. At the experiment’s endpoint, animals were humanely euthanised by CO_2_ inhalation and tissues collected for analysis. Bone marrow cells were harvested by flushing the tibia and femur from both legs with DMEM (Gibco). Bone marrow progenitors were stained for flow cytometry analysis as described below or used to generate BMDM as described above. After red blood cell lysis, washing and counting, 3 million cells were kept for flow cytometry analysis as described below, and the remaining cells were resuspended in DMEM, 10% FBS, 20% L929-conditioned media and seeded to be differentiated into BMDMs over 1 week. To isolate splenocytes, spleens were carefully dissected after euthanasia, collected and kept on ice in RPMI/FBS 0.1% medium. Spleens were then minced into small pieces, gently crushed on a 70μM nylon mesh cell retainer with a syringe and strained with PBS. After a wash, the pellet was resuspended and treated 2 minutes with Red Blood Cell (RBC) Lysis Buffer Hybri-Max™ (Sigma-Aldrich). Splenocytes were then washed again, resuspended in DMEM, 10% FBS, 5% L929-conditioned media and seeded. Cells were allowed to adhere overnight, washed and stimulated with LPS (10 ng/mL) or heat-killed *Mycobacterium tuberculosis* H37Ra (hk-Mtb, 500-1000 μg/mL).

#### Multiparameter flow cytometry analysis of human monocytes

To measure phospho-S6 ribosomal protein activity in human monocytes after β-glucan treatment, PBMCs were isolated as above and resuspended in cRPMI-1640 media supplemented with 10% human AB serum (Sigma-Aldrich) at 2 x 10^6^/mL. Cells were first incubated with metabolic inhibitors for 15 min as indicated. Cells were then stimulated. After the incubation time, cells were washed in PBS. Cells were stained with fixable viability stain ZombieAquaTM (Biolegend) at the concentration of 1:1000 for 15 minutes. Subsequently, samples were washed with PBS and incubated with anti-Dectin-1-PE (clone 15E2, Biolegend) antibody for Dectin-1 surface staining, then washed and fixed 20 minutes with IC Fixation Buffer (Invitrogen). Alternatively, cells were incubated with anti-CD14-APC (clone M5E2, Biolegend), anti-CD16-PE-Cy7 (clone 3G8, Biolegend), anti-HLA-DR-BB515 (clone G46-6, BD Bioscience) at a concentration of 1:100 in flow buffer for 30 minutes at 4°C. All cells were subsequently washed with flow buffer and resuspended 20 minutes with IC Fixation Buffer (Invitrogen) for fixation. Cells were then washed with twice with 1x permeabilization buffer (Invitrogen), incubated at 4°C for 30 minutes with permeabilization buffer containing anti-phospho-S6-PE (Ser235/236) (clone D57.2.2E, Cell Signaling) at 1:200 for intracellular pS6 staining, and finally washed again with with 1X perm buffer and X1 with PBS.Compensation controls were made using UltraComp eBeads™ Compensation Beads (Invitrogen) stained with the appropriate antibodies. Data analysis and was performed using FlowJo software v.7.6 (TreeStar). For combined flow cytometry and imaging of stained cells, cell suspensions were ran on an ImageStream X Mk II (Amnis/ Luminex) cytometer. Data was analyzed and images generated using the ImageStream Data Analysis and Exploration Software (IDEAS).

#### Multiparameter flow cytometry analysis of BMDM

For analysis of intracellular cytokines, dWGP-trained BMDM were treated with 5ng/ml brefeldin-A (Sigma) for the final 4 hours of the stimulation period. Supernatants were discarded and cells were washed with PBS. Cells were incubated on ice with PBS (without calcium and magnesium) for 5 mins and then harvested into FACs tubes. Cells were washed in PBS and stained with fixable viability stain ZombieAquaTM (Biolegend) at the concentration of 1:1000 for 15 minutes. Cells were washed in PBS and incubated with a cocktail of antibodies for 20 mins to stain surface proteins (anti-CD11b – APC (clone M1/70, Biolegend), anti-F4/80 – PeCy7 (clone BM8, Biolegend)). Cells were then washed and fixed 20 minutes with IC Fixation Buffer (Invitrogen). Cells were then washed X2 with 1X perm buffer (Invitrogen), incubated at 4°C for 30 minutes with perm buffer containing anti-TNF – BV421 (clone MP6-XT22, Biolegend). Finally, cells were washed again X1 with 1X perm buffer and X1 with PBS. Cells were resuspended in flow buffer until acquired on the LSR Fortessa with FACSDiva software. Compensation controls were made using UltraComp eBeads™ Compensation Beads (Invitrogen) stained with the appropriate antibodies. Data analysis and was performed using FlowJo software v.7.6 (TreeStar). For quantification of mitochondrial mass and membrane potential, cells were harvested and washed as described. A mitochondrial green (MTG) / Tetramethylrhodamine, methyl ester (TMRM) master mix was prepared in warm DMEM medium. Cells were re-suspended in 200μl of the MTG/TMRM cocktail. Cells were stained for 30 min at 37°C. Cells were then washed X1 with PBS for further staining as described. Cells stained with TMRM and MTG were not fixed and acquired live on an LSR Fortessa cytometer.

#### Multiparameter flow cytometry analysis of mouse bone marrow cells

To analyse HSPC populations in mouse bone marrow after *in vivo* induction of trained immunity, isolated bone marrow cells were resuspended in flow buffer; PBS-1X (Gibco) supplemented with 5% heat inactivated FBS (Gibco) and 0.1% Sodium azide (Sigma). The following flow cytometry staining protocol was applied to all bone marrow samples with up to 3,000,000 cells per sample. Cells were stained with fixable viability stain ZombieAqua™ (Biolegend) at the concentration of 1:500 for 15 minutes. Subsequently, samples were washed with flow buffer and incubated with anti-CD16/32 (Biolegend) at a concentration of 1:100 in flow buffer for 20 minutes at 4°C. The following antibodies were then used for staining Lin- c-Kit+ Sca-1+ cells (LKS), hematopoietic stem cells (HSCs) and multipotent progenitors (MPPs): anti-Ter-119, anti-CD11b (clone M1/70), anti-CD5 (clone 53-7.3), anti-CD4 (clone RM4-5), anti-CD8a (clone 53-6.7), anti-CD45R+ (clone RA3-6B3), anti-Ly6G/C+ (clone RB6-8C5), all biotin-conjugated (all Biolegend) were added at a concentration of 1:50 for 30 minutes at 4°C. Cells were then washed with flow buffer. Streptavidin – APC-Cy7 (Biolegend), anti-c-Kit-APC (clone 2B8, Biolegend), anti-Sca-1-PE-Cy7 (clone D7, eBioscience), anti-CD150 – eFluor450 (clone mShad150, eBioscience), anti-CD48-PerCP-eFluor710 (clone HM48-1, BD Bioscience), anti-CD34-FITC (clone RAM34, eBioscience), anti-Flt3-PE (cloneA2F10.1, Biolegend) were added and incubated at 4°C for 30 minutes. Fluorescence Minus One (FMO) controls were performed using 1,000,000 cells obtained by mixing equivalent volumes of samples coming from the different experimental conditions and stained with the proper antibodies. All cells were subsequently washed with flow buffer and resuspended with IC Fixation Buffer (Invitrogen). Compensation controls were obtained after staining UltraComp eBeads™ Compensation Beads (Invitrogen) with the appropriate antibodies. Cells were acquired on the BD flow cytometer Canto II with FACSDiva software. Data analysis and flow charts were performed using FlowJo software v.7.6 (TreeStar).

### Quantification and statistical analysis

Data shown represents the mean data for experiments carried out on human monocytes/macrophages derived from the number of indicated independent donors, the numbers of indicated animals per groups for *in vivo* studies, or independent BMDM preparations for mouse *in vitro* studies. Pooled data was analysed by Graph Pad Prism and Figures generated in Microsoft Powerpoint. 1-way ANOVA was carried out on experiments where multiple groups were compared to a control stimuli and 2-way ANOVA performed for experiments examining multiple conditions (eg; dose/times), with post-hoc multiple comparison tests to indicate significant differences between treatment groups or conditions as indicated in Figures. For comparisons between 2 groups, student t-tests were performed. ∗ indicates comparisons P<0.05 between untrained & trained cells. # is used to indicate comparisons P<0.05 between control trained cells and trained cells + inhibitors. Source data files for all experiments are deposited in Mendeley Data, V1, doi: https://doi.org/10.17632/kfzjtrmthb.1. RNA-sequencing based gene expression data has been deposited in NCBI GEO & can be accessed under GEO accession number GSE235691. R-code used in this study can be found on GitHub.
