# Supplementary material for: Recognition of yeast β-glucan particles triggers immunometabolic signaling required for trained immunity
Source: iScience. 2024 Jan 26;27(3):109030. doi: 10.1016/j.isci.2024.109030 (PMC10865028; doi:10.1016/j.isci.2024.109030)
Supplement: Document S1. Figures S1–S8 [file mmc1.pdf]

iScience, Volume *27*

Supplemental information

Recognition of yeast β-glucan particles triggers immunometabolic signaling required for trained immunity

Cian J.H. Horneck Johnston, Anna E. Ledwith, Mimmi L.E. Lundahl, Hugo Charles-Messance, Emer E. Hackett, Simon D. O'Shaughnessy, Jonah Clegg, Hannah Prendeville, John P. McGrath, Aaron M. Walsh, Sarah Case, Hollie Austen Byrne, Parth Gautam, Elaine Dempsey, Sinead C. Corr, and Frederick J. Sheedy

SUPPLEMENTAL INFORMATION LEGENDS

Figure S1. Macrophage responses to restimulation in β-glucan trained macrophages, *Related to Figure 1*. **A-C)** Training assays in hMDM trained with BGP or dWGP (10 μg/mL) or tolerized with LPS (10 ng/mL) and restimulated with LPS (10 ng/mL) for the indicated times (0-24h) and TNF production measured (A) or CXCL8 at 3h, TNF at 6h and IL-10 production 24h post-stimulation measured and expressed relative to untrained cells (B), or restimulated with LPS (100 ng/mL), pam3CSK4 (100 μg/mL) or HKCA (10^6cells/mL) and TNF production measured after 6h (C). **D-F)** Training assays in mBMDM trained with BGP or dWGP (100 μg/mL) or tolerized with LPS (10 ng/mL) and restimulated with LPS (10 ng/mL) for the indicated times (0-24h) and TNF production measured (D) or IL6, TNF and IL-10 production 24h post-stimulation measured and expressed relative to untrained cells (E), or restimulated with LPS (100 ng/mL), p(I:C) (5 μg/mL), Zymosan (ZYM) or pam3CSK4 (PAM, 100 μg/mL) and TNF production measured after 24h (F). **G)** hMDM derived from dWGP-trained (10 μg/mL) or LPS-tolerised (10 ng/mL) monocytes were treated with irradiated Mycobacterium tuberculosis H37Rv (iMtb, 500 μg/mL) for 24h and production of the indicated cytokines measured. **H-I)** hMDM derived from dWGP-trained monocytes (10 μg/mL) were infected with Mtb H37Ra (MOI 5 bacteria/cell) and intracellular bacterial survival measured by CFU analysis at the indicated times post-infection (H) or TNF production measured in culture supernatants (I). **J)** Training Assay in dWGP-trained mBMDM restimulated with LPS (10 ng/mL) or pam3CSK4 (100 μg/mL) for 6h. Intracellular cytokine staining for TNF was performed after brefeldin-A treatment (left panel) or ELISA on supernatants derived from cells without brefeldin-A. All data is mean ± sd for n=3 (A-B, D-F, G, J-K), n=4 (C) and n=5 (H-I) or n=2 (J) independent experiments. $^*P<0.05$ or indicated $P \geq 0.05$ determined using multiple comparisons testing following one or two-way ANOVA.

Figure S2. Canonical Dectin-1 signalling does not distinguish β-glucan-induced trained immunity, *Related to Figure 2.* **B)** NFκB-linked SEAP activity in hDectin1a or hDectin-1b-HEK293 reporter cells incubated with the indicated β-glucans (1-100 μg/mL) or LPS (1-100 ng/mL) or left unstimulated (-) for 6h. **C)** Correlation of NFκB-linked SEAP in hDectin-1b-HEK293 reporter cells (Fig. 2A) with Monocyte Training in hMDM (Fig. 1A) for 100 μg/mL β-glucans treatment. **D)** Training Assay in hMDM incubated with solubilized WGP (sWGP, 100 μg/mL) for 1h prior to training with dWGP (1,10 & 100 ug/mL) for 24h. Mature macrophages were restimulated with LPS (10ng/mL, 6h) and TNF production measured. **E)** NFκB-linked SEAP activity in hDectin-1b-HEK293 reporter cells incubated with laminarin or sWGP at the indicated concentrations for 1h prior to treatment with BGP or dWGP at 100 μg/mL for 6h. **F)** hMDM Training Assay from monocytes pre-treated with laminarin or sWGP at the indicated concentrations for 1h prior to training with BGP or dWGP at 100 μg/mL for 24h. Mature macrophages were restimulated with LPS (10 ng/mL, 6h) and TNF production measured. **G)** NFκB-linked SEAP activity hDectin-1b-HEK293 reporter cells incubated with the indicated concentrations of piceatannol (PIC) for 1h prior to treatment with BGP or dWGP at 100 μg/mL for 6h. **H-I)** Production of the indicated cytokine in mBMDM after treatment with the indicated β-glucans (1-100 μg/mL) or LPS (1-100 ng/mL) or left unstimulated (-) for 24h and measured by ELISA. **J)** hMDM Training Assay using supernatants from WGP-trained monocytes (10 μg/mL, 24h) transferred to untrained monocytes (S/N Txt), left for 5-days. Mature macrophages were restimulated with LPS (10 ng/mL, 6h) and TNF production measured. **K)** NFκB-linked SEAP activity in RAW-Blue cells treated with β-glucans or left unstimulated (-) for 6h (E) **L)** TNF production from monocytes incubated with cytochalasin-D (Cyt-D, 10 μM, 1h) and subsequently treated with the indicated b-glucan (10 μg/mL) for 24h. **M)** Training Assay in dWGP-trained mBMDM pre-treated with Cyt-D (10 μM, 1h) and TNF (6h) and IL10 (24h) production measured after LPS restimulation. All data is mean ± sd for n=2 (E-G, L-M), n=3 (A-B, D, H-K) and n=4 (C) independent experiments. $^{*/\#}P<0.05$, ns or indicated $P\geq0.05$

determined using multiple comparisons testing following one or two-way ANOVA or Students t-test (B).

Figure S3. β-glucans drives long-term metabolic reprogramming for training, *Related to Figure 3.* **A)** Extracellular Lactate production in monocytes treated with the indicated β-glucans (1-100 μg/mL) or LPS (1-100 ng/mL) or left unstimulated (-) for 24-72h. **B)** dWGP training assay in mBMDM pre-treated with 2 deoxyglucose (2DG, 25 μM, 1h). IL-6 and IL-10 production were measured 24h after restimulation with LPS (10 ng/mL). **C-E)** Extracellular flux analysis for for mBMDM treated with the indicated β-glucans (100 μg/mL) or LPS (100 ng/mL) or left unstimulated (-) for 24-72h. Glycolytic capacity or maximal respiration rate was calculated and shown in C). Glycolytic traces based on extracellular acidification rates (ECAR) after inhibitor addition (OM; oligomycin and 2DG) shown in D). Respiration traces based on oxygen consumption rates (OCR) after inhibitor addition (OM, FCCP and Rot & AA; rotenone + antimycin-A) shown in E). **F-G)** Flow cytometry gating strategy for mitochondrial activity analysis on mBMDM (top plots) & representative plots for control stimuli (bottom, left -> right; untreated, LPS-stimulated, OM-treatment & FCCP-treatment) with fold changes in mitotracker green (MTG, mitochondrial mass) and tetramethylrhodamine methyl ester (TMRM, mitochondrial activity) signal shown in G). Data is mean ± sd for n=2 (A), n=3 (G) n=4 (B) or n=6 (C-E) independent experiments. $^{*/\#}P<0.05$ determined using multiple comparisons testing following one or two-way ANOVA.

Figure S4. mTOR independent remodelling of TCA during dWGP training, *Related to Figure 4.* **A)** Representative gating strategy used for intracellular staining of monocytes for Phospho-S6 (p-S6) activity. **B)** Representative flow cytometry histogram for p-S6 positivity in monocytes treated with dWGP or sWGP (10 μg/mL) for 2h or left untreated (-). **C)** p-S6 activity in human monocytes after stimulation with the indicated β-glucans (10 μg/mL) or LPS (10 ng/mL) or left unstimulated (-) for 1h. **D)** p-S6 staining in monocytes pre-treated with rapamycin (rapa, 10

nM, 1h) prior to stimulation with dWGP or sWGP (10 μg/mL) or LPS (100 ng/mL) for 2h. **E)** p-S6 staining in monocytes stimulated with dWGP (10 μg/mL) for the indicated times. **F)** dWGP Training Assay in mBMDM pre-treated with Rapa (10 nM). TNF production was measured between 3-24h post-LPS restimulation (10 ng/mL). Data is mean ± sd for n=3 (C-D,F) independent experiments or representative experiments. $^{*}P<0.05$ determined using multiple comparisons testing following one (C-D) or two-way (F) ANOVA.

Figure S5. Phagocytosis of particulate β-glucans, *Related to Figure 5.* **A)** Representative histogram plot of monocytes treated with dWGP (10 μg/mL, 2h) or left untreated (-) and subjected to staining for surface Dectin-1 expression detected by flow cytometry. **B)** Monocytes pre-treated with cytochalasin-D (Cyt-D, 10 μM) or vehicle treated for 1h prior stimulation with dWGP or BGP (100 μg/mL) for 15 min, were immediately fixed and stained for surface Dectin-1 expression measured by flow cytometry. **C)** Monocytes were stimulated with dWGP or BGP (100 μg/mL) for the indicated times and surface Dectin-1 expression detected by flow cytometry. **D)** hMDM Training Assay in monocytes pre-treated with bafilomycin-A1 (Baf-A1, 10 μM) 1h prior to treatment with dWGP (10 μg/mL) or LPS (100 ng/mL). Mature macrophages were restimulated with LPS (10 ng/mL, 6h) and TNF production measured. **E)** TNF production in monocytes pre-treated with PIC (between 4, 10 and 30 μM) 15min prior to stimulation with dWGP (10 μg/mL) for 24h. Data is mean ± sd for n=2 (D) or n=3 (B,E) independent experiments or representative experiments. $^{*}P<0.05$ determined using multiple comparisons testing following two-way ANOVA.

Figure S6. Phagocytosis of intact and pure yeast β-glucan particles drives trained immunity, *Related to Figure 6.* **A)** Schematic illustrating protocol to prepare fractions of solubilized WGP. **B)** NFκB-linked SEAP activity in hDectin1-HEK293 reporter cells incubated with dWGP, sWGP or defined fractions of sWGP F1-F4 (MW ranges indicated below, kDa) at 1, 10 and 100 μg/mL for 6h. **C)** Schematic illustrating protocol to conjugate solubilized WGP to polystyrene particles

(PS). **D)** Sugar content of sWGP preparations after filtration following incubated with 2 µg aminated polystyrene particles (AM-PS) in the presence or absence of conjugating agent 1,1' Carbonyldi-imidazole (CDI). **E)** Lactate production in human monocytes after treatment with 200 µg/mL aminated polystyrene particles (PS) alone or conjugated with sWGP (PS-sWGP), the equivalent amount of sWGP roughly corresponding to 200 µg/ml of sWGP-PS (1 µg/ml) or dWGP (100 µg/ml) or left untreated (-) for 72h. **F)** Schematic illustrating protocol to prepare single particle "dispersible" WGP (dWGP) from aggregated "unsonicated" WGP (uWGP) via sonication. Light micrographs represent monocyte cultures with each preparation. **G)** NFκB-linked SEAP activity inhDectin1-HEK293 reporter cells treated with heat-killed *Candida albicans* (HKCA) at 10^4, 10^5 & 10^6 cells/mL, Zymosan (ZYM), dispersible WGP (dWGP), unsonicated WGP (uWGP) or solubilized WGP β-glucan (sWGP) all at 1,10 & 100 µg/mL, or LPS (1,10 and 100 ng/mL) for 6h. **H)** Extracellular Lactate production in monocytes treated with the indicated WGPs (1-10 µg/mL) or left unstimulated (-) sampled between 24-72h post-treatment. **I)** NFκB-linked SEAP activity in hDectin1a, hTLR2 or hTLR4-HEK293 reporter cells treated with the indicated β-glucans (1-10 µg/mL), LPS (10 ng/mL), PAM3CSK4 (PAM, 100 µg/mL) or left unstimulated (-) for 6h. **J-K)** Training Assay in hMDM (I) or mBMDM (J) trained with Zymosan-depleted (ZYM-d, 10/100 µg/mL). Mature macrophages were restimulated with LPS (10 ng/mL) and TNF production measured relative to untrained cells (-) at the indicated times. Data is mean ± sd for n=5 (B) or n=3 (D-E, G-K) independent experiments. $^{*/\#}P < 0.05$, ns P≥0.05 determined using multiple comparisons testing following two-way ANOVA.

Figure S7. dWGP delivery drives myeloid bone marrow reprogramming, *Related to Figure 7*. C57/BL6-J mice were injected intraperitoneally with dWGP (0.2 & 0.4 mg/mouse), BGP (1 mg/mouse) or vehicle PBS (A-B) or 2mg WGP IP or via oral gavage (C). After 7-days, bone-marrow (BM) was isolated, quantified, stained and analysed by flow cytometry for the indicated populations; c-Lin-1$^-$, c-Kit$^+$, Sca1$^+$ (LKS), Long-Term Hematopoietic Stem Cells (LT-HSC), Short-Term HSCs (ST-HSC), Multipotent Progenitors 1-3 (MPP) or CD150-/CD48- cells. **A)** %

of total LKS cells in bone-marrow cells. **B)** % of LKS subsets in total bone-marrow cells (BM). **C)** TNF production in total splenocytes prepared from mice treated with 0.2 mg dWGP as indicated and stimulated with LPS (10 ng/mL) for 6h. Data is mean ± sd for n=4 (A-C) mice per group. *P < 0.05 determined using multiple comparisons testing following one (A) or two-way (B-C) ANOVA.

Figure S8. dWGP-containing diets reprogramme bone-marrow macrophage responses, *Related to Figure 8*. **A-C)** Mature C57/BL6-J mice were fed control (0 WGP diet) for 2-weeks prior to initiation of feeding diets supplemented with increasing concentrations of dWGP (0, 0.003%, 0.025%, 0.050% per kg chow). Mice were sacrificed 1, 3 or 4-weeks post-dWGP feeding and bone-marrow (BM) examined by flow cytometry. Data is the is relative percentage for the indicated subset in the total LKS population and is mean± sd for n=6-8 mice per group. **C)** Volcano plot of differentially expressed genes in resting BMDM derived from mice fed a control (0 WGP) diet or mice fed 0.025% dWGP for 1-week (n=2 mice per group). *P<0.05 determined using multiple comparisons testing following Mixed Effect Model testing (A-C) or multiple Students t-tests (D). Log_{10} P value thresholds for RNA-sequencing are indicated on plots.

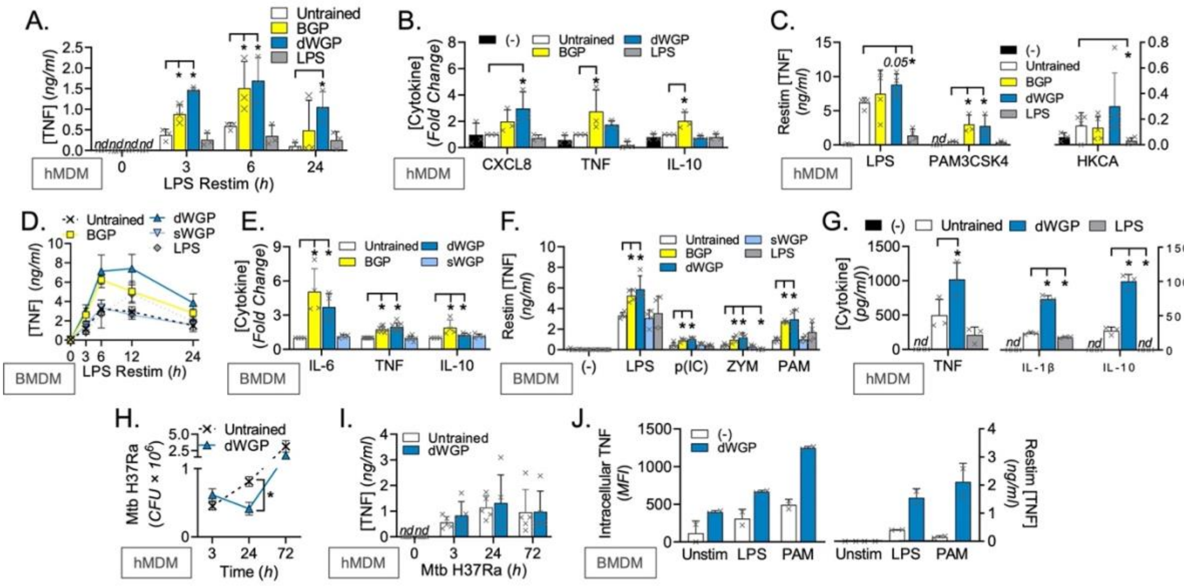
A.
Untrained
BGP
dWGP
LPS
[TNF] (ng/ml)
LPS Restim (h)
hMDM
B.
[Cytokine] (Fold Change)
CXCL8
TNF
IL-10
C.
Restim [TNF] (ng/ml)
0.05
LPS
PAM3CSK4
HKCA
D.
Untrained
BGP
dWGP
sWGP
LPS
BMDM
E.
IL-6
F.
(-)
p(IC)
ZYM
PAM
G.
[Cytokine] (pg/ml)
IL-1β
H.
Mtb H37Ra (CFU × 10^6)
Time (h)
I.
Mtb H37Ra (h)
J.
Intracellular TNF (MFI)
Unstim
nd

Figure S1

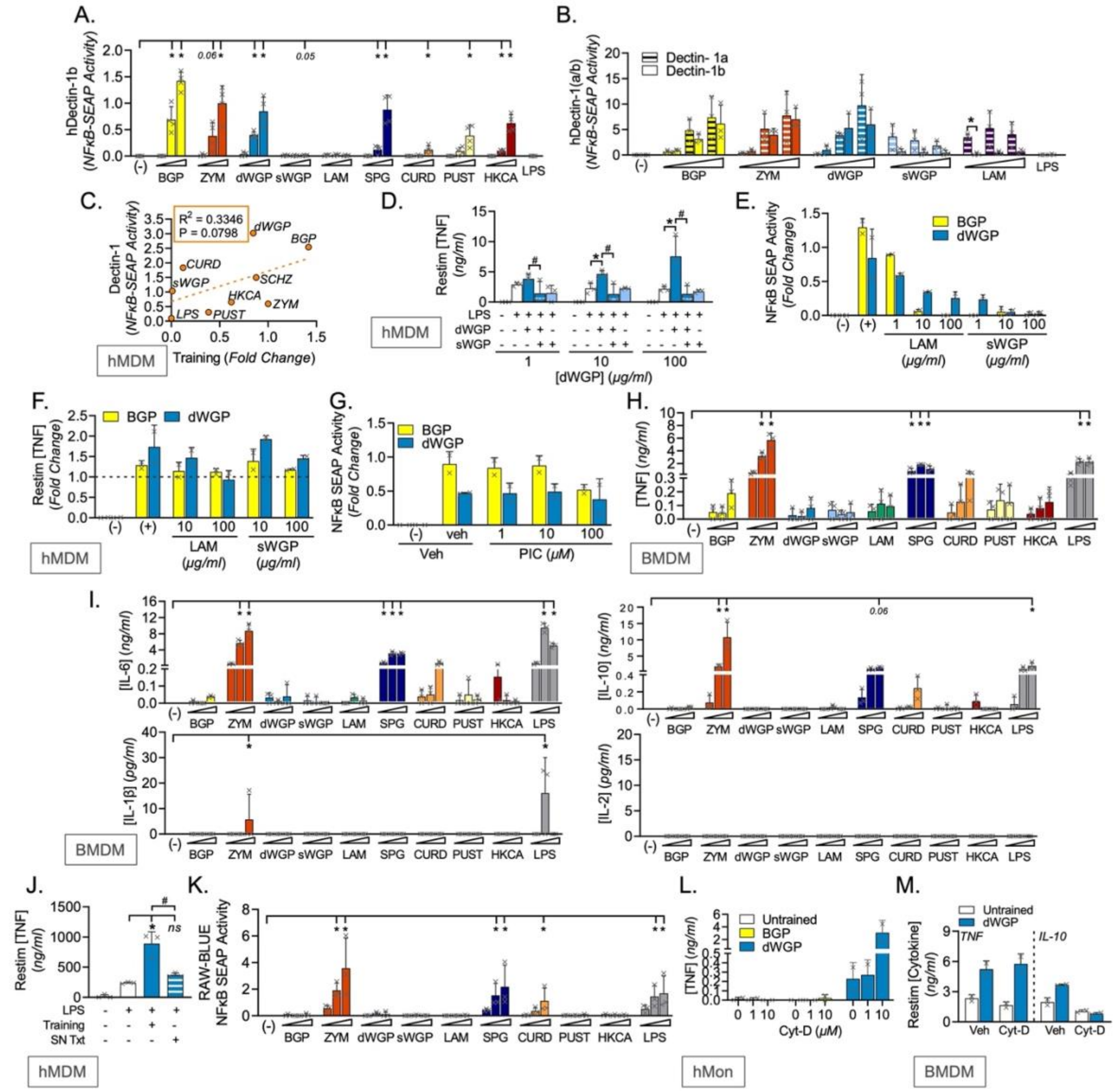

Figure S2

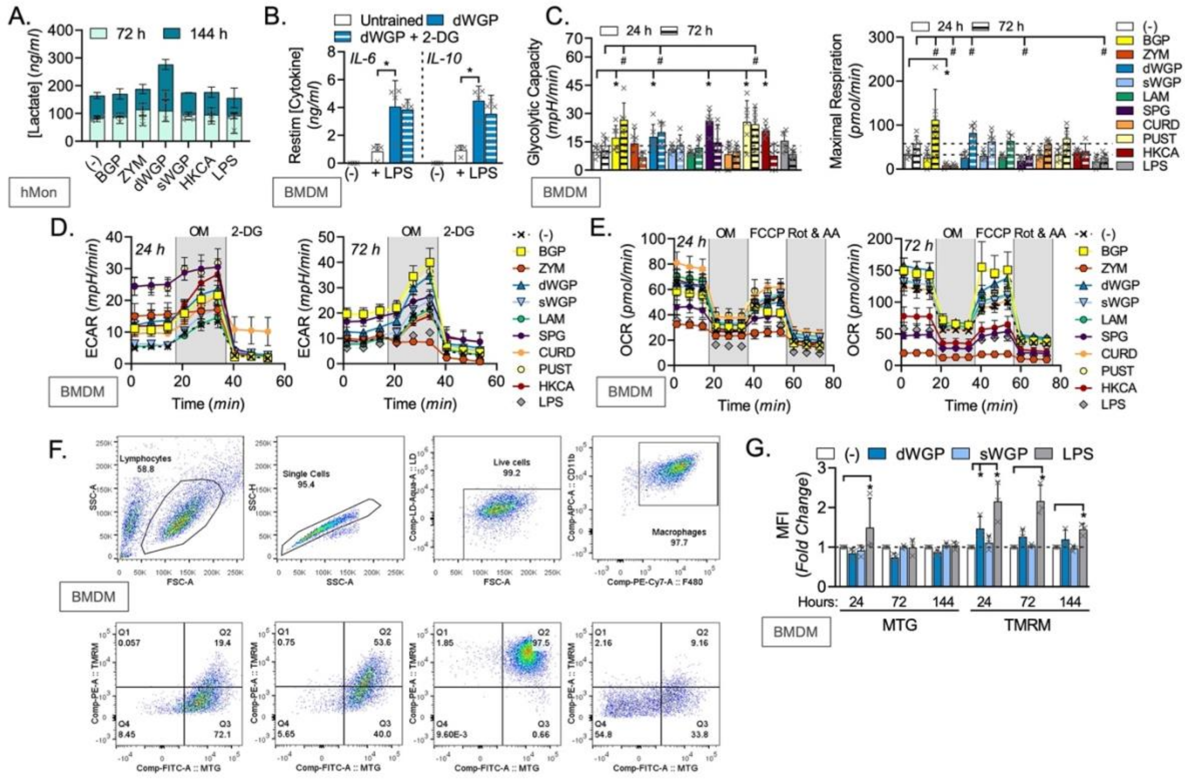

Figure S3

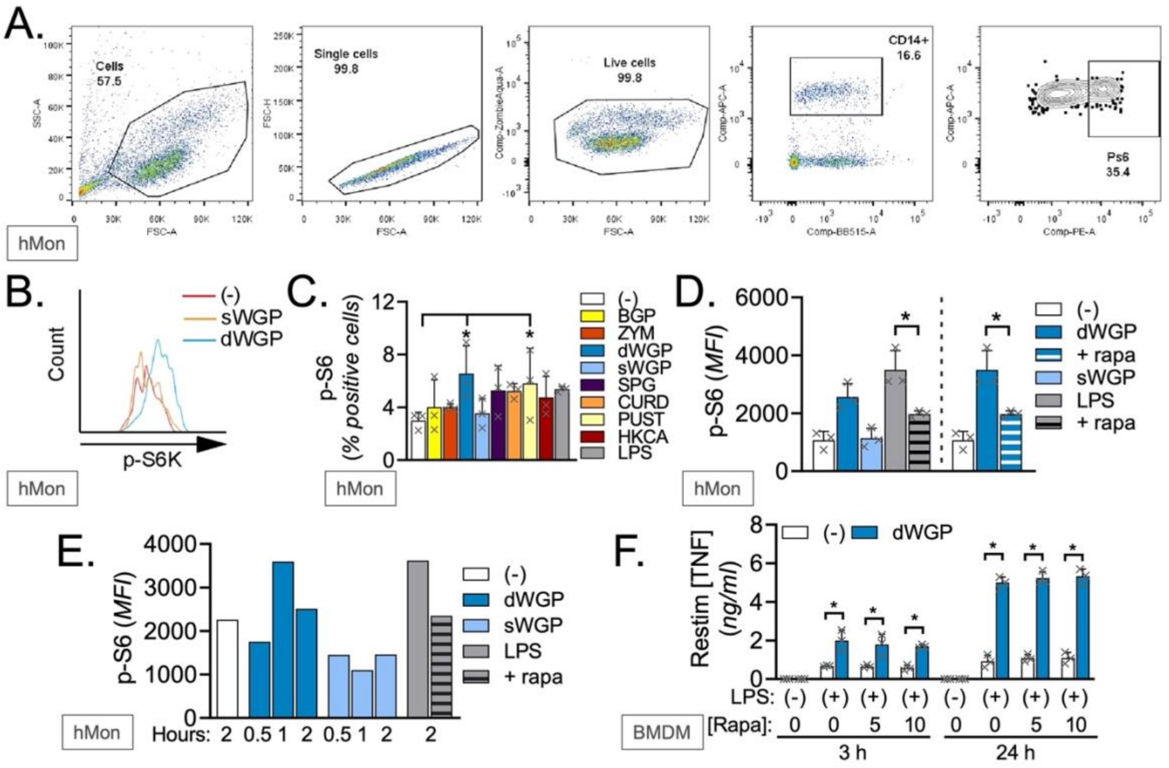
A.
Cells 57.5
Single cells 99.8
Live cells 99.8
CD14+ 16.6
Ps6 35.4
hMon
B.
(-)
sWGP
dWGP
Count
p-S6K
hMon
C.
p-S6 (% positive cells)
(-)
BGP
ZYM
dWGP
sWGP
SPG
CURD
PUST
HKCA
LPS
hMon
D.
p-S6 (MFI)
(-)
dWGP
+ rapa
sWGP
LPS
+ rapa
hMon
E.
p-S6 (MFI)
(-)
dWGP
sWGP
LPS
+ rapa
hMon
Hours: 2 0.5 1 2 0.5 1 2 2
F.
(-) dWGP
Restim [TNF] (ng/ml)
LPS: (-) (+) (+) (+) (-) (+) (+) (+)
[Rapa]: 0 0 5 10 0 0 5 10
3 h
24 h
BMDM

Figure S4

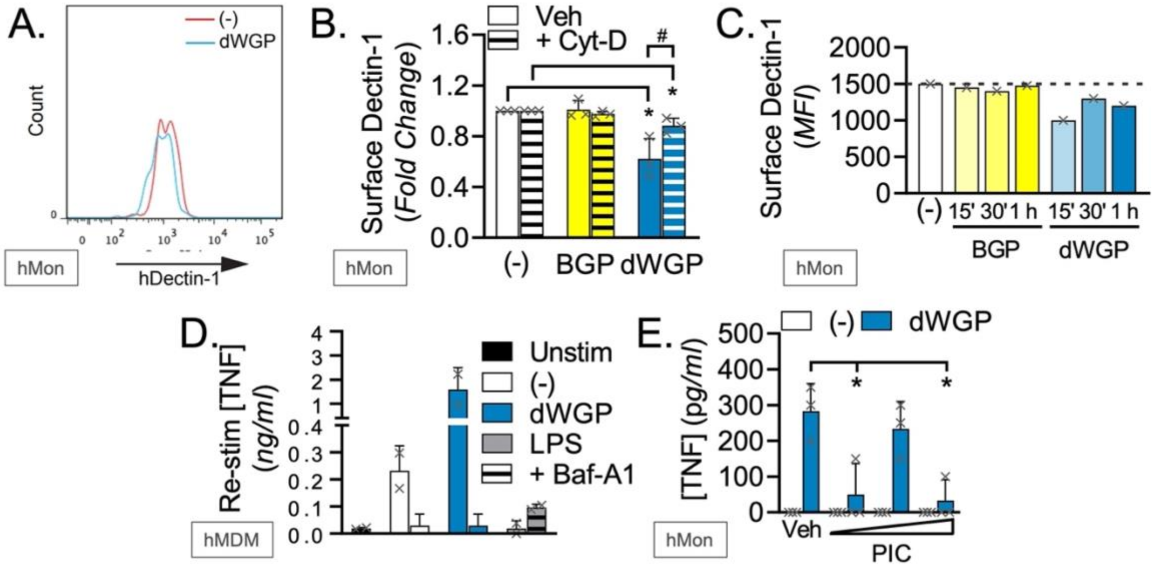
A.
(-)
dWGP
Count
hDectin-1
hMon
B.
Veh
+ Cyt-D
Surface Dectin-1
(Fold Change)
(-)
BGP
dWGP
hMon
C.
Surface Dectin-1
(MFI)
(-)
15' 30' 1 h
BGP
15' 30' 1 h
dWGP
hMon
D.
Re-stim [TNF]
(ng/ml)
Unstim
(-)
dWGP
LPS
+ Baf-A1
hMDM
E.
(-)
dWGP
[TNF] (pg/ml)
Veh
PIC
hMon

Figure S5

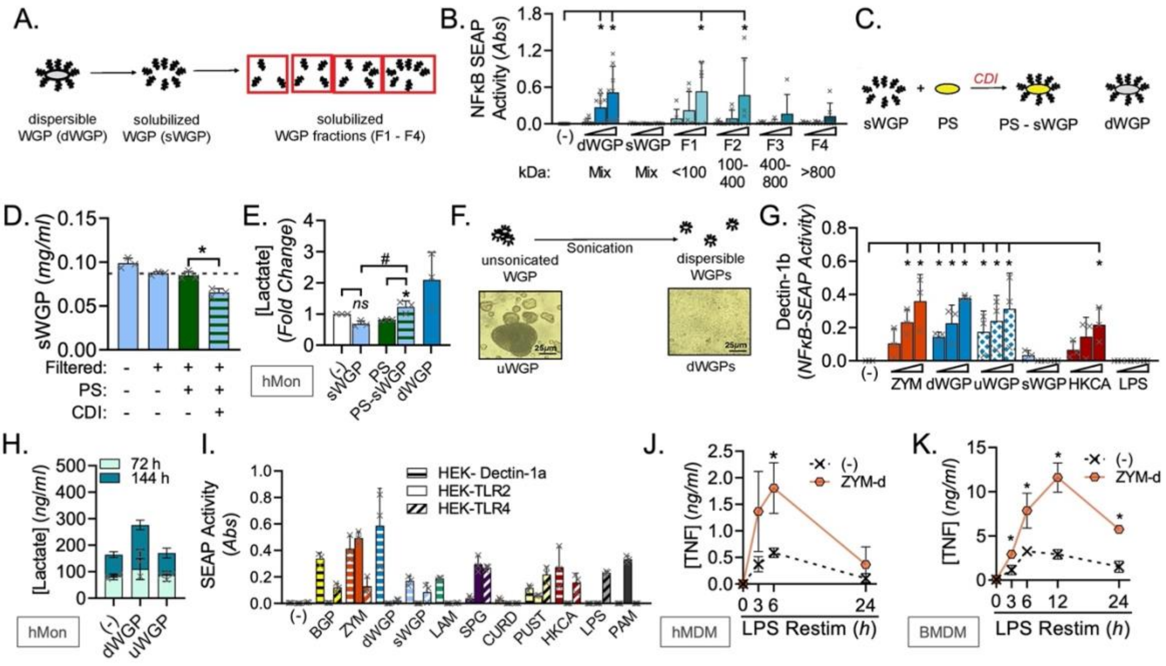
A.
dispersible WGP (dWGP)
solubilized WGP (sWGP)
solubilized WGP fractions (F1 - F4)
B.
NFκB SEAP Activity (Abs)
(-) dWGP sWGP F1 F2 F3 F4
kDa: Mix Mix <100 100-400 400-800 >800
C.
sWGP PS CDI PS - sWGP dWGP
D.
sWGP (mg/ml)
Filtered:
PS:
CDI:
E.
[Lactate] (Fold Change)
hMon
(-) sWGP PS PS-sWGP dWGP
F.
unsonicated WGP
Sonication
dispersible WGPs
uWGP
dWGPs
G.
Dectin-1b (NFκB-SEAP Activity)
(-) ZYM dWGP uWGP sWGP HKCA LPS
H.
[Lactate] (ng/ml)
72 h
144 h
hMon
(-) dWGP uWGP
I.
SEAP Activity (Abs)
HEK- Dectin-1a
HEK-TLR2
HEK-TLR4
(-) BGP ZYM dWGP sWGP LAM SPG CURD PUST HKCA LPS PAM
J.
[TNF] (ng/ml)
(-)
ZYM-d
hMDM
LPS Restim (h)
K.
[TNF] (ng/ml)
(-)
ZYM-d
BMDM
LPS Restim (h)

Figure S6

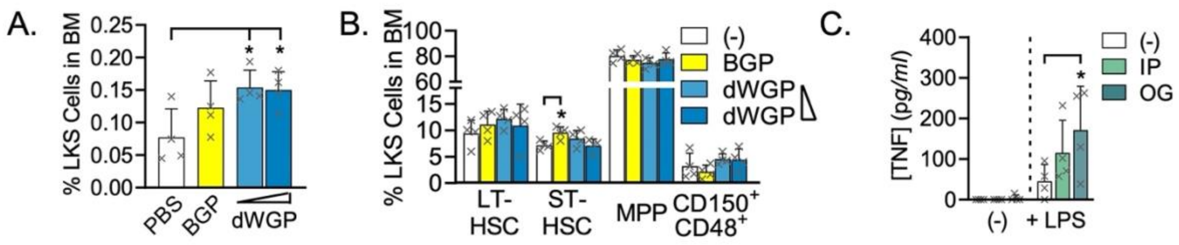
A.
% LKS Cells in BM
0.25
0.20
0.15
0.10
0.05
0.00
PBS
BGP
dWGP
B.
% LKS Cells in BM
100
80
60
15
10
5
0
(-)
BGP
dWGP
dWGP
LT-HSC
ST-HSC
MPP
CD150+ CD48+
C.
[TNF] (pg/ml)
400
300
200
100
0
(-)
IP
OG
(-)
+ LPS

Figure S7

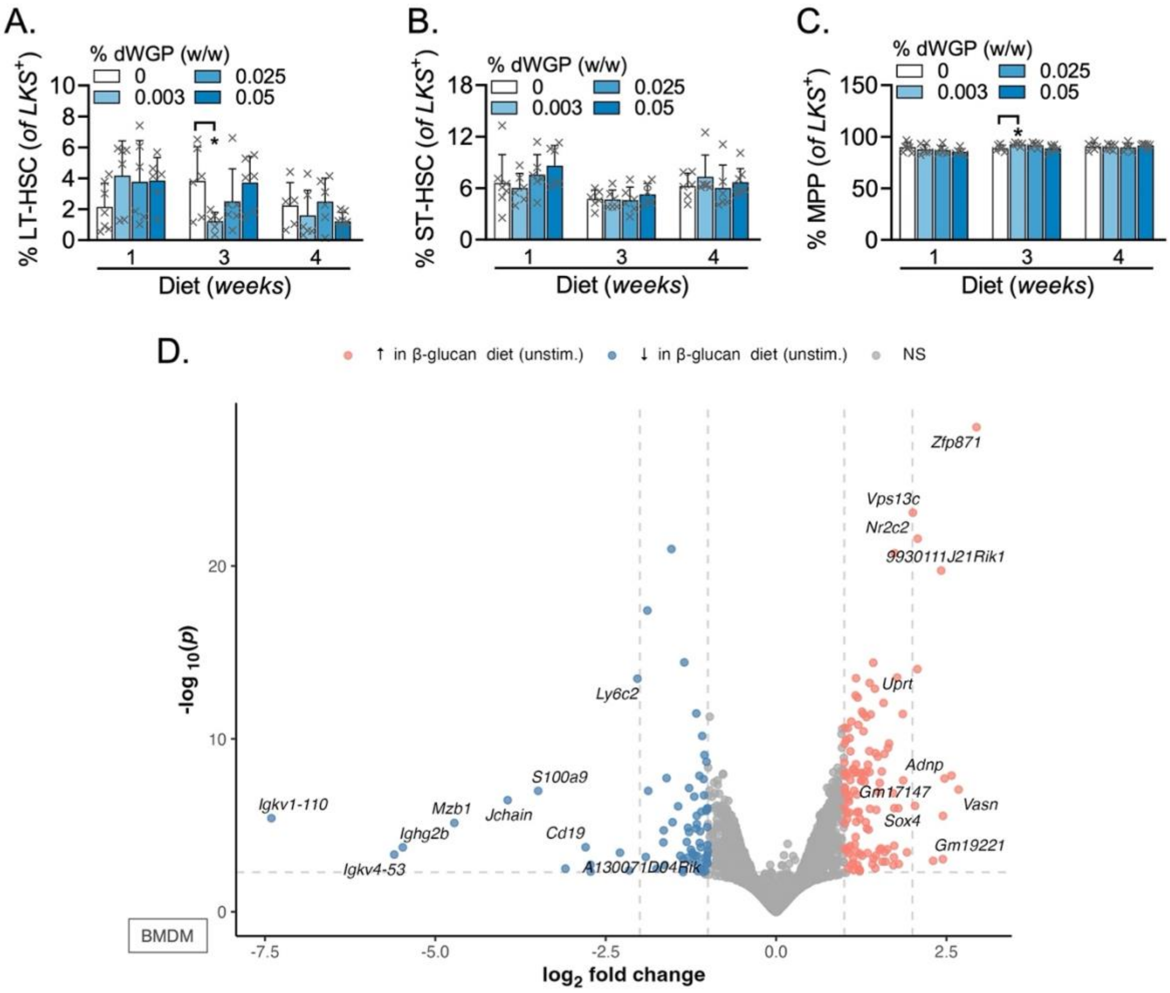

A.
% dWGP (w/w)
0
0.003
0.025
0.05
% LT-HSC (of LKS+)
Diet (weeks)
B.
% ST-HSC (of LKS+)
C.
% MPP (of LKS+)
D.
↑ in β-glucan diet (unstim.)
↓ in β-glucan diet (unstim.)
NS
-log 10(p)
log2 fold change
BMDM
Zfp871
Vps13c
Nr2c2
9930111J21Rik1
Uprt
Adnp
Gm17147
Vasn
Sox4
Gm19221
Ly6c2
S100a9
Igkv1-110
Mzb1
Jchain
Ighg2b
Cd19
Igkv4-53
A130071D04Rik

Figure S8
